# Concentration, source identification, and exposure risk assessment of PM2.5-bound parent PAHs and nitro-PAHs in atmosphere from typical Chinese cities

**DOI:** 10.1038/s41598-017-10623-4

**Published:** 2017-09-04

**Authors:** Di Liu, Tian Lin, Jabir Hussain Syed, Zhineng Cheng, Yue Xu, Kechang Li, Gan Zhang, Jun Li

**Affiliations:** 1 State Key Laboratory of Organic Geochemistry, Guangzhou Institute of Geochemistry, Chinese Academy of Sciences, Guangzhou, 510640 China; 2 State Key Laboratory of Environmental Geochemistry, Institute of Geochemistry, Chinese Academy of Sciences, Guiyang, 550081 China

## Abstract

Sixteen parent PAHs and twelve nitro-PAHs were measured in PM2.5 samples collected over one year (2013–2014) at nine urban sites in China. During the sampling period, concentrations of individual nitro-PAHs were one or two orders of magnitude lower than their parent PAHs. Typical seasonal variations in parent PAH concentrations, which increased 10- to 80- fold in winter compared to summer, were observed in this study. Conversely, the mean atmospheric concentrations of nitro-PAHs were similar in all four seasons, with the exception of 9-nitroanthracene (9n-Ant). Compared to other nitro-PAHs which were secondary formation products, 9n-Ant had a higher concentration and made up a larger proportion of total nitro-PAHs. Positive matrix factorization results indicated that 9n-Ant sources included biomass burning (20%), vehicle exhaust emissions (43%), and secondary formation (30%). Overall, the elevated concentrations of parent PAHs observed in winter correlated with the contribution from coal combustion at all sites, especially in north China (>80%). The contribution of secondary formation products to total nitro-PAHs was measured during the summer, and was especially high in the larger cities such as Shanghai (84%), Beijing (76%), Guangzhou (60%), and Chengdu (64%), largely due to the summer concentrations of parent PAHs were markedly lower than in winter.

## Introduction

The Industrial Revolution and the resulting increase in energy consumption have resulted in major changes in environmental quality. In modern China, air pollution is a serious problem, and major cities have experienced an increasing number of hazy days in recent years^1^. An increasing number of urban residents across the country have realized that air pollution has become a serious threat to their health, and that PM2.5 is one of the most significant potential hazards, because it can be deposited much more deeply in the lungs than coarser particles when inhaled. PM2.5 acts as a carrier for various toxic organic compounds, which further increases the risk associated with human exposure. As one of the most studied families of toxic organic compounds present in PM2.5, polycyclic aromatic hydrocarbons (PAHs) are of great environmental concern due to their carcinogenic and mutagenic properties^2, 3^. About 500 PAHs and related compounds have been detected in the air, and among them are some of the most toxic compounds known, such as benzo[a]pyrene (BaP)^4^. The US EPA has classified sixteen parent PAHs as priority pollutants based on their toxicity, potential for human exposure, frequency of occurrence at hazardous waste sites, and the extent of information available about them^5^. In recent years, nitro-PAHs (e.g., dinitropyrene) have become of particular concern because they possess higher direct mutagenicity and carcinogenicity than their parent PAHs, which are indirect mutagens that depend on metabolic activation^2, 6^. For example, the mutagenic activity of 1,8-dinitropyrene is three orders of magnitude higher than that of BaP, which is considered one of the most toxic PAHs. Thus, although nitro-PAH concentrations are generally lower than parent PAHs in the atmosphere, increasing attention has been paid to these pollutants in recent years^7, 8^.

PAHs are mainly released from human activities. Fossil fuel combustion, aluminum and iron/steel production, coal and coke production, biomass burning, traffic emissions, and oil spills are all sources of exposure^9^. In China, industrial processes and the use of fossil fuels have accounted for slightly more than one quarter of the emissions of the sixteen parent PAHs, while the burning of wood and other biomass has contributed more than half^10, 11^. Different types of combustion have yielded different distributions of PAHs, both in terms of the relative amounts of individual PAHs and which isomers are produced, meaning that the composition of mixed PAHs may be useful as an indicator of their source. Numerous source studies measuring diagnostic ratios of parent PAHs in different cities have identified multiple and varied sources of poor air quality^12, 13^. However, these ratios should be used with caution, because the degradation products of compounds may vary with time in the atmosphere^14^. Nitro-PAHs are formed directly from combustion processes such as those in diesel and gasoline engines, as well as by oxidants in the atmosphere through both gas-phase and heterogeneous reactions of PAHs, and are also useful as tracers for the identification of air pollution sources and photochemical pathways^15, 16^. These pathways include reactions of the parent PAH with OH or NO_3_ radicals in the gas phase, and with N_2_O_5_ or HNO_3_ when the parent PAH is in the aerosol phase^17^. Furthermore, the relative contribution of daytime (OH radical) and nighttime (NO_3_ radical) reactions in the gas phase can be determined by the formation ratio of 2-nitrofluoranthene (2n-Flu) to 2-nitropyrene (2n-Pyr)^18^. A wide variety of nitro-PAHs have been detected in diesel exhaust particles in urban environments, with the main recognized primary sources of nitro-PAHs being vehicle exhaust emissions and *in situ* formation. Extremely high concentrations of 9-nitroanthracene (9n-Ant) compared to other nitro-PAHs have been observed in sugar cane burning regions, indicating that biomass burning is an important source of 9n-Ant in addition to diesel exhaust^19^. Both 3-Nitrophenanthrene (3n-Phe) and 9-nitrophenanthrene (9n-Phe) may be formed in the atmosphere by gas-phase reactions of phenanthrene, although they have also been identified in exhaust emissions from trucks^20, 21^. The main nitro-PAH present in diesel exhaust is 1-nitropyrene (1n-Pyr), which can also be formed via heterogeneous nitration reactions of particle-associated pyrene^21^. However, the sources (via primary emission or secondary formation) of these PAH derivatives in the atmosphere are not yet documented quantitatively.

More than 80% of particle-bound PAHs are associated with PM2.5, and PAH concentrations in particulate matter are highly dependent on these fine particles^22, 23^. Thus, studies of PAHs in PM2.5 can provide useful tools for environmental monitoring and source identification. In this study, 12 nitro-PAHs and 16 parent PAHs were measured in PM2.5 samples collected over one year at nine urban sites across China (Beijing, Shanghai, Guangzhou, Nanjing, Wuhan, Chengdu, Taiyuan, Lanzhou, and Xinxiang, Table S1). The objectives of this study were: (1) to determine ambient levels of nitro-PAHs and parent PAHs in urban PM2.5 and to identify their possible sources based on spatial and temporal concentration variations (using principal component analysis, PCA), and (2) to apportion the sources of PAHs in PM2.5 using positive matrix factorization (PMF) modeling. This study provides quantitative information on the sources of PAHs, allowing a more comprehensive assessment of the risk of human exposure to PAHs from various sources in these cities. Finally, our results will serve as a basis for regulatory and management standards to be used by the environmental authorities responsible for monitoring air quality.

## Results

### Parent PAH concentrations in PM2.5

**Figure 1:**
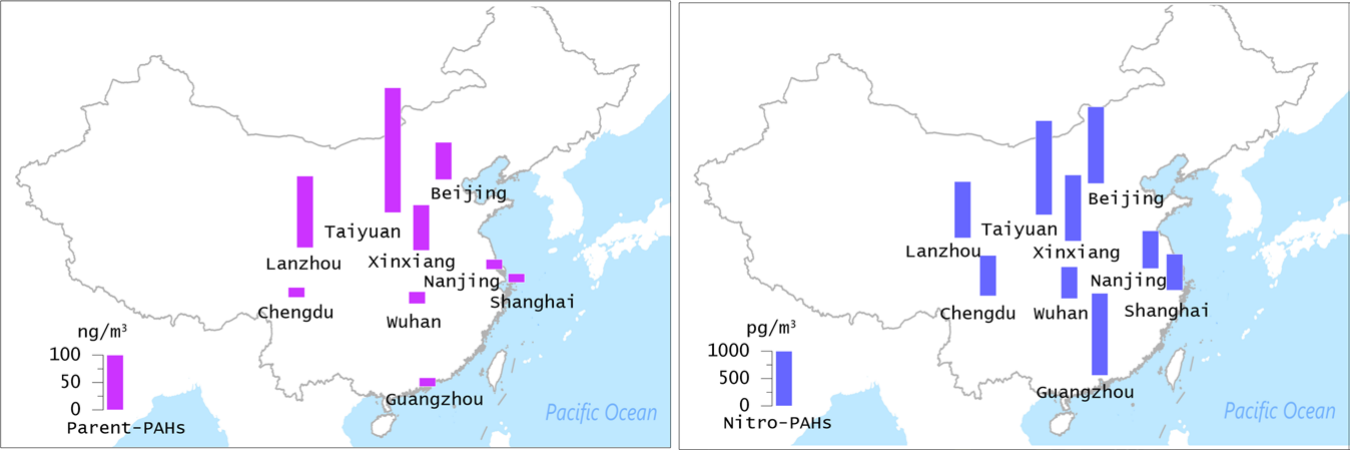
Spatial distributions of the concentration of parent PAHs and nitro-PAHs in PM2.5 from 9 urban cities. (the background map was made using surfer 8.0 by one co-author).

The annual mean concentrations of parent PAHs and nitro-PAHs at all sites are shown in Fig. 1, and detailed data on individual PAHs are given in Table S2 (Table S3 shows list of compounds analysed and their abbreviations). Aside from Nap, Ac, and Ace, all compounds were detected in 100% of the samples. The mean and standard deviation of the concentration of total parent PAHs (∑PPAH) at all nine sites were 65 ± 120 ng/m^3^. The lowest ∑PPAH (3.0 ng/m^3^) was detected at the Nanjing site on 4 July 2014, and the highest, 580 ng/m^3^, occurred at the Lanzhou site on 4 January 2014. As was expected, the ∑PPAH concentrations varied over a wide range temporally and spatially (3.0–580 ng/m^3^). The concentrations found in this study were generally comparable to those previously reported in urban areas of China^24–26^. The most dominant individual PAH found in this study was Flu, followed by BbF, Pyr, and Chr. High concentrations of Flu, BbF, Pyr, and Chr, which account for a large proportion of the total parent PAHs, have been commonly observed in urban air particulates in northeast Asia, as well as from several background reference sites in China, especially during the winter^15, 27, 28^. Based on the number of aromatic rings, the parent PAHs can be subdivided into five groups: 2-, 3-, 4-, 5-, and 6-ring PAHs. The profiles of these PAH classes in each urban area are shown in Figure S1. The concentration of 2-ring PAH (Nap) ranged from ND to <1%, while 3-ring PAHs ranged between 6 and 9%. The percentages of 4-, 5-, and 6- PAHs were 34–51%, 28–36% and 12–21%, respectively. PAHs with high molecular weight (4–6 rings) tend to be associated with particle phase. Parent PAHs with 4–6 rings were predominant in PM2.5 in this study (>90%), presumably because of the important contributions of coal combustion (Chr and BbF are source markers^29^), household biomass fuel burning (Flu, Pyr, and BbF^30^) and gasoline emissions (IP and BghiP^31^). Thus, although a wide range of concentrations were observed for ∑PPAH, the parent PAH profiles exhibited a relatively uniform distribution among the different sites, suggesting that parent PAHs at most urban sites came from similar sources, with slightly different proportions.

### nitro-PAH concentrations in PM2.5

The concentrations of total nitro-PAHs (∑NPAH) were 0.37–6.5 (1.1 ± 0.9) ng/m^3^ for all samples in this study. The concentrations of ∑NPAH fell into the narrow range of 0.37–6.5 ng/m^3^, much lower variation than ∑PPAH (3.0–580 ng/m^3^). These observations were consistent with previous measurements of nitro-PAHs in the air in China, which are typically 10–100 times lower than those of parent PAHs^32, 33^. The most abundant nitro-PAH was 9n-Ant, followed by 2-nitronaphthalene (2n-Nap) and 1-nitronaphthalene (1n-Nap). The concentrations of these three components were one or two orders of magnitude higher than other nitro-PAHs. The predominance of 9n-Ant, 2n-Nap, and 1n-Nap in atmospheric particles has been previously reported in Mexico City, Mexico (with a proportional contribution fluctuating between 50–70%)^34^, the Marseilles area in the south of France^35^, and urban and rural areas in northern China^36^. Due to the differences in target compounds among different studies and the limited data available for nitro-PAHs, the mean concentrations (presented in pg/m^3^) of individual compounds were used for comparison (Table S4). For example, the mean concentration of the most abundant reported nitro-PAH, 9n-Ant, was 560 ± 790 pg/m^3^ in this study, which was in the same order of magnitude as concentrations that have been measured in the atmosphere of northern China^36^, but significantly higher than in the Marseilles area, France (56.6–227.7 pg/m^3^)^35^, Los Angeles, USA (32.3 pg/m^3^)^37^, the urban area of Copenhagen, Demark (63 pg/m^3^)^38^, and Mexico City, Mexico (45.7 pg/m^3^)^34^. The concentrations of 1n-Nap and 2n-Nap were 100 ± 35 pg/m^3^ and 220 ± 65 pg/m^3^ in this study, respectively, which were comparable to measurements in the atmosphere of northern China^36^, but remarkably lower than those in a sugar cane burning region, Brazil (nd-1800 for 2n-Nap)^39^, and rural households in China^30^. Compounds with low molecular weight predominately exist in the gaseous phase, e.g. 1n-Nap and 2n-Nap, which were the near detection limits in airborne particles from typical industrial sites in south China and São Paulo, Brazil^39, 40^. Conversely, four high-molecular-weight nitro-PAHs are the most-frequently reported PAHs in urban particulate matter: 3-nitrofluoranthene (3n-Flu), 1-nitropyrene (1n-Pyr), 7-nitrobenz[a]anthracene (7n-BaA) and 6-Nitrochrysene (6n-Chr). The 3n-Flu concentrations in this study (33 ± 19 pg/m^3^) were significantly lower than in the urban areas of Santiago, Chile (597 pg/m^3^)^41^, Tokyo, Japan (261.3 pg/m^3^)^42^, and were similar to those in Cordoba, Argentina (25–27 pg/m^3^) and Madrid, Spain (21 pg/m^3^)^43, 44^. The 1n-Pyr values in this study (32 ± 37 pg/m^3^) were much lower than in Tokyo, Japan (106.3 pg/m^3^) and Copenhagen, Demark (127 pg/m^3^)^15, 38^, but were comparable to Madrid, Spain (41 pg/m^3^)^43^. The mean concentration of 7n-BaA in this study (29 ± 10 pg/m^3^) was comparable with that in Cordoba, Argentina (23–28 pg/m^3^), but lower than in urban Hanoi, Vietnam (41–214 pg/m^3^), which has a high density of traffic^44, 45^. The mean concentration of 6n-Chr, one of the most toxic nitro-PAHs, was 3.0 ± 0.9 pg/m^3^ in this study. These concentrations were similar to other urban centers in China, but lower than in Madrid, Spain, Cordoba, Argentina and Mexico City, Mexico^34, 43, 44^. Although its concentrations were not very high, 6n-Chr is regarded as one of the most carcinogenic nitro-PAHs, and thus might be harmful to human health at low concentrations. Compared with other sites around the world, the concentrations of low molecular weight nitro-PAHs (9n-Ant, 1n-Nap and 2n-Nap) were slightly higher in this study, while high molecular weight nitro-PAHs (3n-Flu, 1n-Pyr, 7n-BaA and 6n-Chr) were detected at a low to moderate levels. Thus, unlike parent PAHs, atmospheric levels of individual nitro-PAHs did not follow the trend of ∑NPAH and instead varied widely among sites. This suggests that concentrations of individual nitro-PAHs depend on more complicated factors including the emission source, gas/particle partitioning, weather conditions (temperature and solar radiation) and the presence of other atmospheric pollutants (CO, NO_X_, and O_3_)^46^.

### Spatial variations of PAHs in PM2.5

The spatial variations in nitro-PAHs and parent PAHs at all sites are shown in Fig. 1. The annual mean of ∑PPAH was highest in Taiyuan (210 ± 200 ng/m^3^), followed by Lanzhou (130 ± 200 ng/m^3^), Xinxiang (82 ± 76 ng/m^3^), Beijing (67 ± 120 ng/m^3^), Wuhan (21 ± 19 ng/m^3^), Nanjing (19 ± 18 ng/m^3^), Chengdu (18 ± 13 ng/m^3^), Guangzhou (16 ± 15 ng/m^3^), and Shanghai (14 ± 28 ng/m^3^). The mean values of ∑PPAH in northern China (Taiyuan, Xinxiang, Lanzhou, and Beijing) were significantly higher than in southern China (Chengdu, Wuhan, Guangzhou, Nanjing, and Shanghai) because the northern cities depend on coal rather than petroleum, natural gas, or hydroelectric power for industrial and domestic activities. Burning of coal emits much higher levels of PAHs than combustion of petroleum products or natural gas^29^. Several high concentrations of ∑PPAH observed in north China suggest that the sampling sites were under the direct influence of a strong point source nearby, especially during the wintertime. Annual mean ∑NPAH concentrations were highest in Taiyuan (1.7 ± 1.2 ng/m^3^), followed by Guangzhou (1.6 ± 0.82 ng/m^3^), Beijing (1.4 ± 1.5 ng/m^3^), Xinxiang (1.2 ± 0.76 ng/m^3^), Lanzhou (1.1 ± 0.51 ng/m^3^), Chengdu (0.73 ± 0.18 ng/m^3^), Shanghai (0.68 ± 0.17 ng/m^3^), Nanjing (0.68 ± 0.18 ng/m^3^), and Wuhan (0.57 ± 0.33 ng/m^3^). ∑NPAH concentrations had a slightly different spatial variation than ∑PPAH. In addition to the traditional coal-burning cities in north China (Taiyuan, Xinxiang, and Lanzhou), the larger cities in south China (Guangzhou and Shanghai) also had relatively high ∑NPAH concentrations. The ∑NPAH concentrations were relatively uniform in these cities, suggesting they came from a mix of sources rather than coal combustion. It is likely that the nitro-PAHs present in the atmosphere in these larger cities originated from vehicle emissions or *in situ* secondary formation within urban areas^33, 36^.

### Seasonal varations of PAHs in PM2.5 by PCA

**Figure 2:**
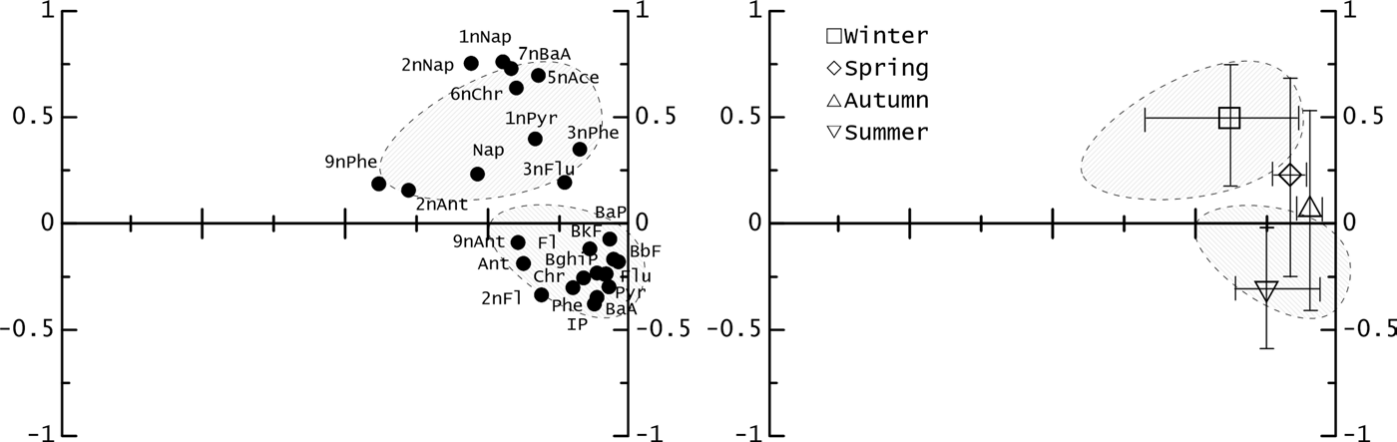
PCA loadings (left) and scores (right) plots for the first two components of all PAHs in PM2.5 from 9 urban sites.

PCA provides a simple and clear discrimination with minimal information loss to determine the distribution of PAHs in the samples and to explain their possible sources. In this study, PCA was performed on all PAH data as one unit. PCA results are usually represented by loading and score plots (Fig. 2). PC1, explaining 55% of the variance, had a high positive loading for parent PAHs (except Nap) and a moderately positive loading for 2-Nitrofluorene (2n-Fl) and 9n-Ant. PC2 accounted for 17% of the total variance and was driven by most nitro-PAHs, with an especially high positive loading on 1n-Nap, 2n-Nap, 7n-BaA, and 6n-Chr. Both 2n-Fl and 9n-Ant were located on the negative side of the PC2 axis, suggesting the sources of these compounds differed from other nitro-PAHs, but were similar to the parent PAHs. Based on the scores plot, we can clearly observe the seasonal variation among samples, but there was no obvious spatial clustering. Samples collected during the winter season fell into the lower-right corner, suggesting a PAH composition with relatively high proportions of 4- to 6-ring parent PAHs. Samples collected in the summer were positioned at the top of the x-axis, and were enriched in nitro-PAHs and relatively depleted in 4- to 6-ring parent PAHs, indicating that a general increase in the proportions of 1n-Nap, 2n-Nap, 7n-BaA, and 6n-Chr occurred from winter to summer. The samples collected in spring and autumn were characterized by a moderate loading of two PCs, with these observations falling between the two clusters.

**Figure 3:**
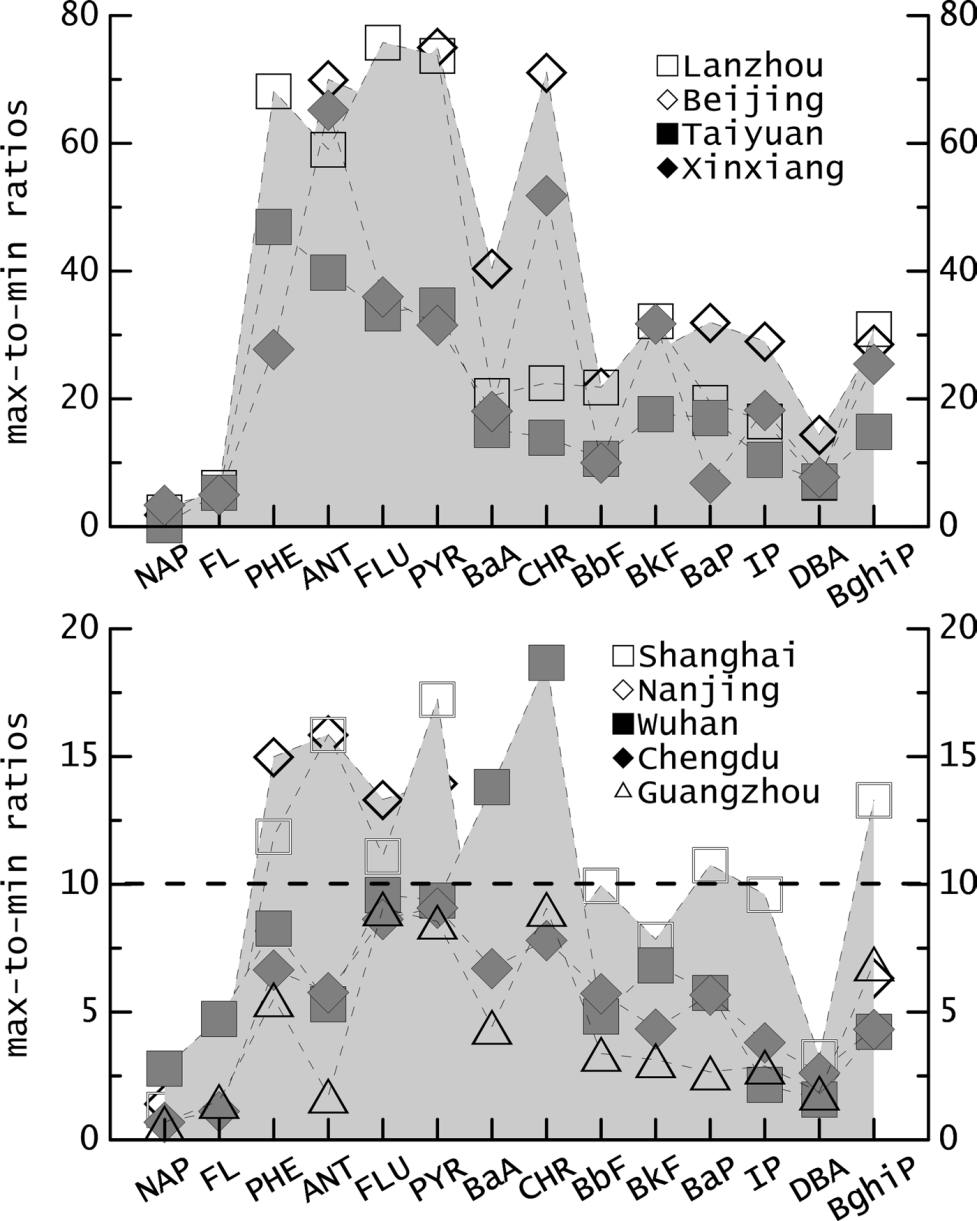
Variations between winter and summer in the concentrations of individual parent PAHs (indicated by max-to-min ratio) in PM2.5 at 9 urban sites.

We noted significant seasonal variation in the concentrations of PAHs, especially parent PAHs, in this study. Without exception, seasonal variations in parent PAH concentrations exhibited their highest concentrations in winter and lowest concentrations in summer at all sites. In this study, the variations between winter and summer in the concentrations of individual compounds (indicated by max-to-min ratios) at each site were used to determine potential sources. The greatest max-to-min concentration ratios were observed in Lanzhou, which had a 73-fold increase for Pyr, 75-fold for Flu, 68-fold for Phe, and 59-fold for Ant. Similarly, in Beijing the compounds Pyr, Chr, Ant, and Phe all increased by more than 50-fold (Fig. 3). These compounds (mainly 3- and 4-ring PAHs) are often considered good markers of coal combustion, in particular low-temperature coal combustion used for residential heating and cooking in winter^29^. Compared with 3- and 4-ring PAHs, relatively low max-to-min ratios were observed for 5- and 6-ring PAHs in those cities. Our observations suggest that the contribution from vehicle exhaust was stable between the non-heating and heating periods of the year. The increasing trend in parent PAHs from summer to winter was also observed in Wuhan, Nanjing, and Shanghai, but these cities had lower max-to-min ratios (<20 fold) than in north China. The lowest max-to-min ratios were observed in Guangzhou and Chengdu, where the concentrations increased by a factor of less than 10 for all compounds (Fig. 3). Overall, there were low levels of parent PAH pollution with no significant spatial differences in summer, whereas high concentrations were observed in winter with notable spatial differences across China. The local winter PAH emissions in north China were higher than those in south China due to intensive industrial activity and domestic coal combustion in north China. The international metropolitan cities with low heating requirements, Shanghai and Guangzhou, also had significant seasonal variations, despite the local regulation of PAHs under the more stringent Air Pollution Prevention and Control Law. The higher concentrations observed in winter in these megacities were likely induced by long-range atmospheric transport^28^. Additionally, calm winds, a vertical temperature inversion, low temperature, low atmospheric mixing height, and low levels of precipitation are common in winter under stagnant conditions, and are not conducive to the diffusion and dilution of airborne PAHs in the larger cities. Under these atmospheric conditions, pollutant dispersion is low, leading to a shift from the gaseous to particulate phase, which can accumulate and create higher concentrations of PAHs in PM2.5^47^. Conversely, during summertime, high precipitation, wind speed, and temperature, along with enhanced photodecomposition of PAHs, result in increased dispersion and thus a decrease in the concentration of PAHs^48^.

Differences between winter and summer were not significant for most nitro-PAHs. The majority of max-to-min ratios were close to 1, with the exceptions of 2n-Fl and 9n-Ant. Different patterns of ∑NPAH/∑PPAH ratios were observed. One pattern was characterized by high values (>0.1) in summer, in particular >0.3 in Guangzhou; While the lower ratios (<0.03) observed in winter. In the wintertime, although levels of nitro-PAHs were similar to summer, their relative abundance decreased because of an increase in parent PAHs from direct sources such as coal combustion, biomass burning, and traffic emissions. Higher parent PAH concentrations had no correlation with concentrations of nitro-PAHs, suggesting that secondary formation rather than primary emission was the major contributor to nitro-PAHs in this study. Furthermore, secondary formation through atmospheric nitration of particle-bound nitro-PAHs was a minor contributor compared to gas-phase photochemical formation, and temperature inversion had a limited direct influence on the enhancement of nitro-PAHs in winter^49^. Accordingly, several extreme observations of high nitro-PAH concentrations occurred in summer, indicating that high temperature and strong radiation conditions are favorable for the secondary formation of nitro-PAHs in the atmosphere via gas-phase photochemical reactions^46^.

## Discussion

### Source apportionment of PAHs by PMF

Traditional PAH source identification based on PAH composition or the ratios of selected PAH concentrations can distinguish between petrogenic and pyrogenic origins, but is unable to determine the contributions of specific source categories. Some multivariate receptor models have been constructed based on an analysis of the correlations between the measured concentrations of chemical species, assuming that highly correlated compounds were derived from similar sources. One commonly-used multivariate receptor model is PCA, which we successfully applied to infer different sources of PAHs. However, PCA is not an optimal tool for quantifying source contributions. The PMF model was developed in order to address this problem, and was also successfully used to determine PAH sources in this study.

Each profile obtained using PMF in this study was compared with PAH source markers and a reported emissions inventory for China. Coal combustion is a dominant source of PAHs in Chinese cities. Several previous studies have found a major contribution of PAHs from coal combustion^50, 51^. Some studies have gone further, separating coal combustion sources into domestic coal combustion (at low temperature) and industrial processes (at high temperature), e.g., coke production and coal-fired power plants. Biomass burning is the largest source of PAHs, and wildfires are the major type of biomass burning in China^9^. However, biomass is burned for heating and cooking mainly in rural areas; thus, the contribution of biomass burning to PAHs in urban areas varied significantly with geographical and meteorological conditions^11^. The contribution of vehicles to PAH emissions was smaller than coal combustion or biomass burning. However, emissions from motor vehicles are an important source of PAHs in urban areas. As vehicle densities continue to increase, the relative contribution of motor vehicles to total PAH emissions is expected to increase in the near future^52^.

**Figure 4:**
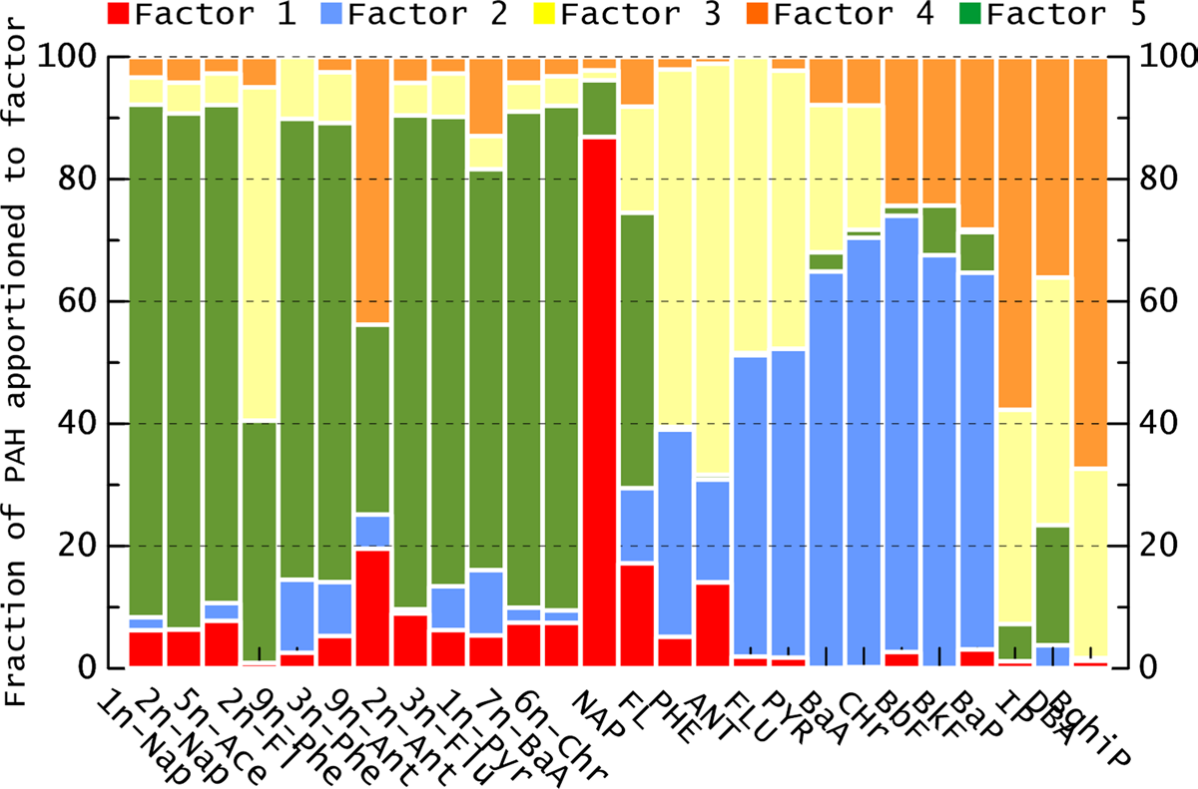
Five-factor loadings by PMF analysis from PAHs data of parent PAHs and nitro-PAHs in PM2.5 from 9 urban cities.

Five distinct sources of PAHs were identified in this study (Figures S2–S5). A profile of each factor is shown in Fig. 4 and Figure S3. Factor 1 was heavily weighted on NAP, and to a lesser extent on Fl and Ant. High emissions of 2- and 3-ring PAHs are associated with biomass burning. Although Flu, Pyr, and Chr have significant loadings in the biomass burning source profile, similar profiles have often been observed in sediments rather in air. Thus, factor 1 was considered to be a source representing biomass burning. We observed an important contribution of 9n-Ant (>20%) from biomass burning. Chuesaard *et al*. (2014) reported that high concentrations of 9n-Ant were not observed at a roadside site, and it has been used a marker for biomass burning during the dry season in addition to diesel exhaust in Thailand^53^. Ding *et al*. (2012) reported one of the highest concentrations of 9n-Ant, and large contribution to total nitro-PAHs in particulate matter collected in rural Chinese homes that use biomass (firewood and corn residue) as fuel^30^. Higher levels of Ant may be produced by biomass combustion at low temperatures, which then produces more 9n-Ant^30, 54^. A 9n-Ant/1n-Pyr ratio of more than 10 indicates a strong contribution from biomass burning^53^. In this study, a contribution of 9n-Ant from biomass burning, designated as a 9n-Ant/1n-Pyr ratio greater than 10, was observed in 36% of samples (n = 51), which was consistent with the PMF results.

Factor 2 was predominately composed of BbF, BkF, BaP, Chr, and BaA with moderate loadings on Pyr and Flu. Chr is a marker for coal combustion, while Pyr, BaA, Chr, BbF, BkF, and BaP are predominant in coal combustion profiles^55, 56^. This source has been identified as a marker of high-temperature coal combustion derived from coking, power plants, and the steel and iron industries^54, 56^. In this study, a Flu/(Flu + Pyr) ratio > 0.5 and a BaA/(BaA + Chr) ratio > 0.35 were consistent with these PAHs sourced mainly from coal combustion^57^.

Factor 3 had the profile of coal combustion at low temperature, i.e., for residential heating and cooking. Although factor 3 contained more abundant IP and BghiP than literature profiles of coal combustion, the IP/(IP + BghiP) ratio > 0.5 still indicated coal burning as a major source in this factor. Besides, relative high loading from IP and BghiP was possible associated with PAHs emitted from cooking oil fumes during residential cooking. Thus, it was considered to represent a source as residential emission.

Factor 4 mainly consisted of the 6-ring PAHs IP and BghiP, with smaller amounts of BaP, BbF, and BfK. Several tunnel and dynamometer studies have been used to identify compounds that are specific to mobile sources. BghiP and IP have been considered tracers of automobile emissions in traffic tunnels, roadsides and urban environments. BghiP was enriched in traffic tunnels along with BaP^58^. Ma *et al*. (2016) found that BghiP has a positive correlation with total PAHs in PM2.5, and attributed the primary source of PAHs in PM2.5 in Hong Kong to vehicles^59^. An IP/(IP + BghiP) ratio < 0.5 was indicative of petroleum combustion of 6-ring PAHs in this factor^57^. Thus, this factor was considered to signify vehicle emissions. A large contribution to this factor was made by 9n-Ant. Several reports have suggested that 9n-Ant is generated from diesel exhaust; e.g., Feilberg *et al*. (2001) reported that the dominant source of 9n-Ant at an urban site in Denmark was direct emission from diesel vehicles, whereas its dominant source at a semirural site was atmospheric secondary formation^38^. Unlike other nitro-PAHs, significant positive correlations between 9n-Ant and factor 4 may confirm that a portion of the 9n-Ant measured in this study was mainly emitted from vehicles.

Factor 5 is highly loaded on most nitro-PAHs, with moderate loadings on 2n-Fl and 9n-Ant. It suggests that these nitro-PAHs have completely different sources from their parent PAHs. The presence of 1n-Pyr in ambient air samples is a nitro-PAH marker for diesel exhaust because it is widely known to be present in diesel exhaust^35^. All of 3n-Flu, 7n-BaA, and 6n-Chr are directly emitted from diesel engines, and they are also indicative of pollution from diesel vehicles^35, 38^. However, Alam *et al*. (2015) noted that after most nitro-PAHs are emitted from vehicles, they are influenced or altered during atmospheric mixing^16^. Nitro-PAHs are released into the environment as a result of direct emissions from incomplete combustion processes and are also formed *in situ* in the atmosphere by gas-phase oxidation and radical (OH or NO_3_) reactions of PAHs, e.g. 3n-Phe and 9n-Phe. 1n-Nap and 2n-Nap were among the most abundant nitro-PAHs in PM2.5, largely formed from the gas-phase OH radical reaction of naphthalene^40^. 2n-Flu is only produced from the gas phase reaction between Flu and NO_2_ initiated by OH during the day and initiated by ON_3_ during the night. 2n-Flu and 2n-Pyr were observed in background area where primary sources are absent and can be as a typical secondary formation source in the atmosphere. In this study, 2n-Flu and 2n-Pyr were hardly detected likely due to the short time of the reaction before sampling. Even so, factor 5 was identified as a source profile representing secondary formation in the atmosphere.

We found no correlation between 9n-Ant and most nitro-PAHs in this study, supporting direct emissions as the main source of 9n-Ant, whereas the other compounds were derived primarily from secondary formation in the atmosphere. The secondary formation products in this study were closely linked to atmospheric photochemical reactions in the gas phase of the atmosphere. However, the formation of 9n-Ant is likely to occur through heterogeneous reactions of nitrating agents with Ant during combustion processes (biomass burning or vehicle emissions) associated with aerosols rather than via gas-phase photochemical reactions in the atmosphere. Ant may be produced at higher levels during combustion processes and become immediately available for heterogeneous reactions to form 9n-Ant. It is very likely that 9n-Ant emitted directly from diesel engines adheres readily to particles. This would result in co-emission with the parent PAH; thus, a portion of the 9n-Ant by heterogeneous reactions was grouped into direct emissions by PMF. The distribution of 9n-Ant was also an exception among observations of nitro-PAHs in northern Mexico City, with the investigators noting that some of the 9n-Ant originated from diesel engine exhaust, while some was formed through heterogeneous nitration reactions between anthracene and the nitrate radical^34^. Similar aspects of this discussion apply equally to 2n-Fl. Burning biomass and low-temperature coal combustion emitted large amounts nitrogen oxides and parent fluorene and 2n-Fl was present in these source emissions as a result of nitration of fluorene.

**Figure 5:**
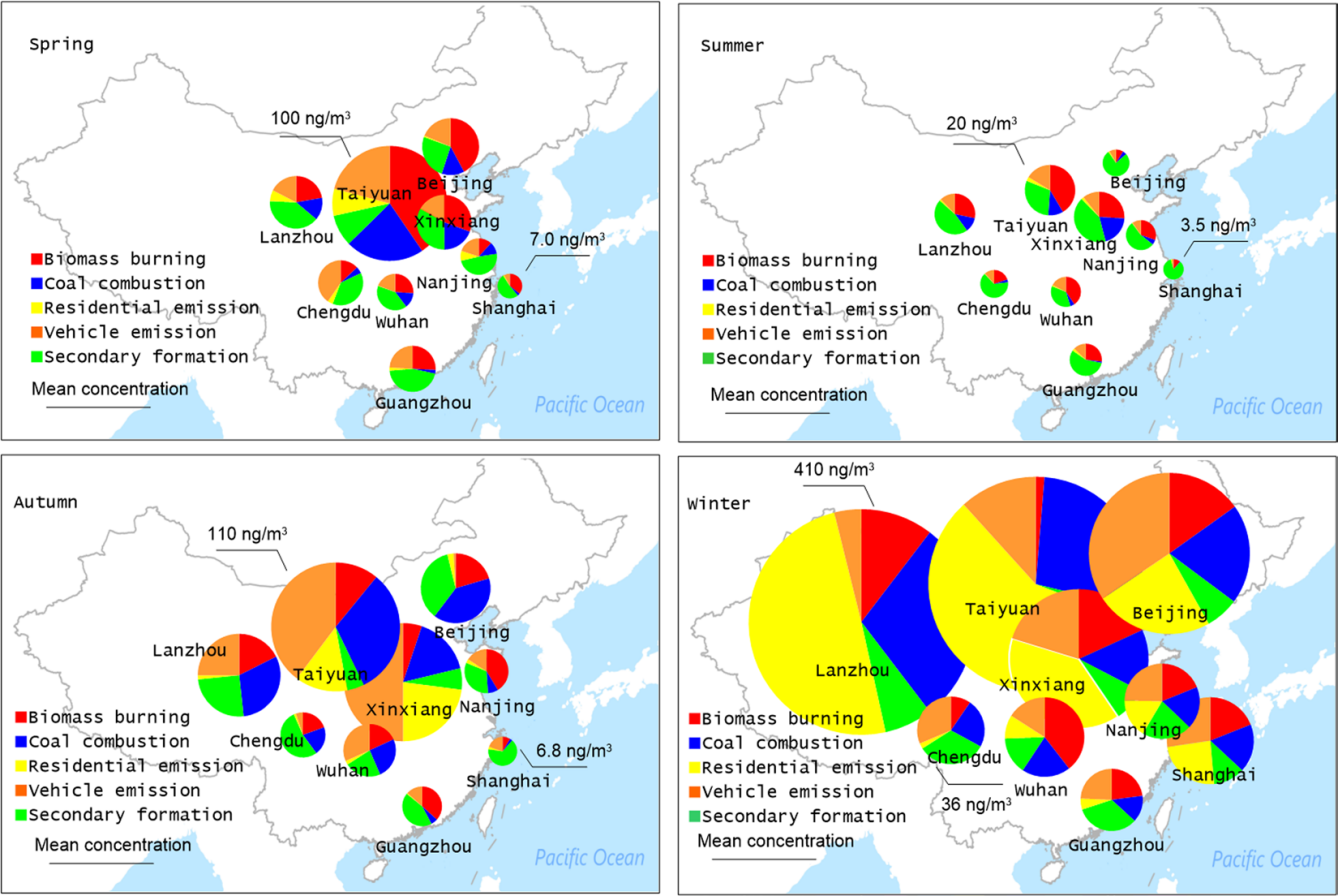
Contributions of the five sources to the total PAHs in PM2.5 at 9 urban cities over four seasons. (the background map was made using surfer 8.0 by one co-author).

The pie chart in Fig. 5 shows the contributions of each source to total PAHs over four seasons in all cities. This figure suggests that seasonal variations in sources differ among regions of China, in addition to the large differences between summer and winter. An important contribution of secondary formation source to PAHs in PM2.5 was observed during the summer, accounting for more than 50% of total PAHs on average, and even more in large cities such as Shanghai (84%), Beijing (76%), Guangzhou (60%), and Chengdu (64%). These results are consistent with particle-associated nitro-PAHs mainly being produced by photochemical reactions between PAHs in the gas phase initiated by hydroxyl radicals during the day and nitrate radicals at night. Thus, more nitro-PAHs were produced during the summer due to high levels of gaseous pollutants undergoing photochemical reactions^35, 46^. The remaining contributions were mainly from biomass burning and vehicle emissions. Additionally, a smaller contribution of PAHs from coal combustion (factors 2 and 3) was observed in Taiyuan, Xinxiang, and Lanzhou, accounting for less than 20% of total PAHs.

In winter, an elevated contribution from high-temperature coal combustion was observed at all sites, which was consistent with the increase in coal consumption to meet electrical demand in both north and south China in winter. Besides, the sources of PAHs in these cities fell into three groups. Group 1 included Taiyuan, Lanzhou, and Xinxiang, which are traditional coal-based industrial cities in north China. More than 60% of the PAH emissions in these cities were from coal combustion (factors 2 and 3), which reached 85% and 79% in Taiyuan and Lanzhou, respectively. Group 2 included Beijing, Nanjing, and Shanghai, in which the contributions from the five sources were nearly equivalent. Group 3 included Guangzhou, Wuhan, and Chengdu. Contributions from residential coal combustion accounted for less than 10% in Group 3 cities, possibly due to the low demand for residential heating in winter. The remaining contributions were primarily from biomass burning (in Wuhan) and secondary formation (in Chengdu and Guangzhou).

### Risk assessment

**Figure 6:**
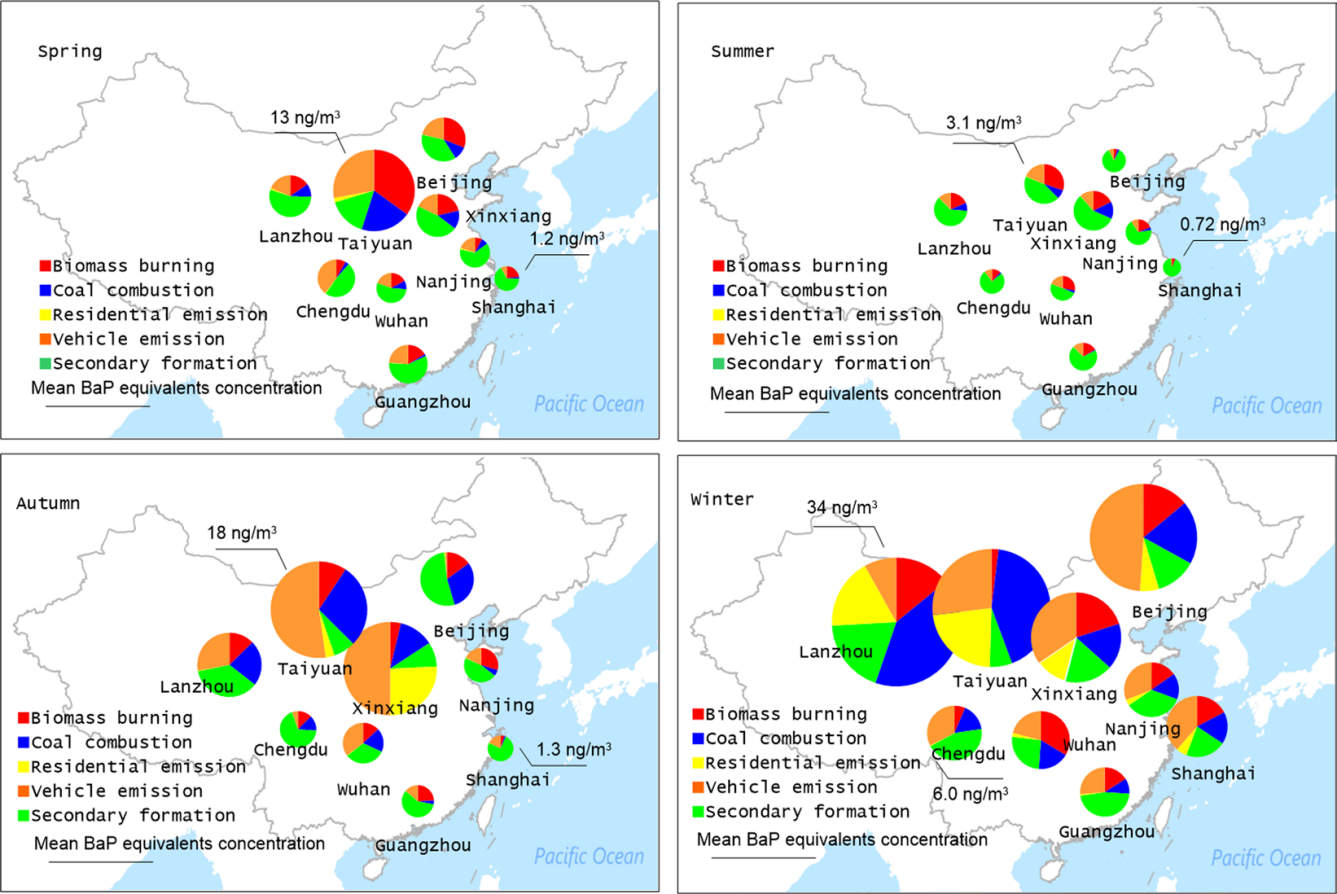
Contributions of the five sources to the BaPeq concentrations of the total PAHs in PM2.5 at 9 urban cities over four seasons. (the background map was made using surfer 8.0 by one co-author).

The results of risk assessment for human exposure in terms of total PAHs are summarized in Fig. 6. Among the individual PAHs, the risk from 16 parent PAHs was assessed according to TEFs published by the US EPA in 1994 and other studies (Table S3). A health risk assessment for nitro-PAHs and PAHs was also conducted in the cities utilizing TEFs. Total BaP equivalents (BaPeq) for the study period were in the range of 0.73–34 ng/m^3^. In spatial terms, Taiyuan (23 ± 23 ng/m^3^) had the highest BaPeq, followed by Lanzhou (12 ± 18 ng/m^3^), Xinxiang (8.3 ± 6.9 ng/m^3^), Beijing (8.3 ± 13 ng/m^3^), Chengdu (2.9 ± 2.0 ng/m^3^), Wuhan (2.7 ± 2.6 ng/m^3^), Guangzhou (2.4 ± 2.0 ng/m^3^), Nanjing (2.4 ± 1.6 ng/m^3^), and Shanghai (1.7 ± 3.0 ng/m^3^). These results are consistent with other observations in China, including Taiyuan (28 ng/m^3^)^60^, Xi’an (17 ng/m^3^)^61^, Guangzhou (11 ng/m^3^)^32^, Beijing (10 ng/m^3^)^33^, but significantly higher than values in Western developed countries, which have more stringent emission standards. Although the atmospheric concentrations of ∑NPAH were lower than those of ∑PPAH and risks were calculated using only four nitro-PAHs, the mean value of the risk from nitro-PAHs accounted for 1.6% (range, 0.6–5.7%) of total PAHs. Because abundant nitro-PAHs such as 9n-Ant, 1n-Nap, 2n-Nap, and 3n-Phe, were not determined and were excluded from calculations due to the absence of TEF values, the values presented in this study underestimated risks. Thus, toxicological data on nitro-PAHs are urgently needed to enable accurate risk assessments.

The spatial and seasonal variations in mean BaPeq concentrations were consistent with total PAH concentrations (Figs 5 and 6). BaPeq was significantly correlated with total PAHs (R^2^ = 0.95). Risk estimates for total PAH exposure were one order of magnitude higher in winter than in summer. Using the samples from winter as an example, Taiyuan, and Lanzhou were classified as high-risk regions, and the major exposure sources in these regions were related to coal burning, including residential and high-temperature combustion. Moderate-risk regions included Beijing, Xinxiang, and Shanghai. Vehicle emissions were the major source of human exposure to PAHs in these cities, followed by high-temperature coal combustion and biomass burning. During the study period, Guangzhou, Wuhan, and Chengdu were found to be low-risk regions. The major exposure sources of PAHs in these urban areas included residential emission and vehicle emissions. Because high-temperature coal combustion and vehicle emissions generated high molecular weight PAHs with a relatively high toxicity, they were identified as the major cause of increased toxicity in PM2.5. For example, PAHs from high-temperature coal combustion contributed 17–30% of the total PAH concentrations in north China (Lanzhou, Taiyuan, Xinxiang, and Beijing) in winter, while the toxicity ascribed to this source reached 47%. In summer, the concentrations of parent PAHs were several-fold lower than in winter, which increased the relative proportion of nitro-PAHs. Secondary formation provided the dominant contribution of BaPeq, especially in Beijing, Shanghai, Guangzhou, and Chengdu, where it contributed over 80% of the total PAH toxicity. These results suggest that it is necessary to control coal combustion in order to reduce the health risk from PAHs in wintertime, while also paying more attention to preventive measures for nitro-PAH pollution.

In the present work, the application of PMF receptor model merging with parent PAH signatures may be an alternative method to obtain the quantitative information on the sources of nitro-PAHs. It should be noted that unlike the gas-phase photochemical reactions in the atmosphere, a portion of nitro-PAH (e.g. 9n-Ant and 2n-Fl in this study) by heterogeneous reactions during the combustion process was grouped into direct emissions by PMF. Overall, this work suggests that the implementation of PMF receptor model in source apportionments and risk assessments would be beneficial, though further investigation is still required.

## Methods

### Sampling information

PM2.5 samples were collected in nine cities (Beijing, Shanghai, Guangzhou, Nanjing, Wuhan, Taiyuan, Chengdu, Lanzhou, and Xinxiang) across China during four seasons (Fig. 1). The sampling locations were in densely inhabited districts within each city, with the sampling sites located on rooftops approximately 15~20 m above ground level. Details of the sampling sites are summarized in the Supporting Information (SI) (Table S1). Each sample was collected over 24 h on a 20.3 × 25.4-cm pre-combusted (5 h at 450 °C) Whatman quartz microfiber filter (QFF) using high-volume samplers operated at 300 L/min. Four seasonal sampling intervals were conducted, 22 Oct. 2013 to 13 Nov. 2013, 30 Dec. 2013 to 20 Jan. 2014, 30 Mar. to 20 Apr. 2014, and 26 Jun. to 24 Aug. 2014. The 4 samples in one season were selected one out of every four samples in order, and total of 16 PM2.5 samples were collected and measured for PAHs and nitro-PAHs at each site.

### Extraction and instrumental analyses

Filters were weighed before and after sampling to determine the mass of PM2.5. The extraction and instrumental analyses have been described elsewhere. Briefly, five PAH surrogates (naphthalene-d8, acenaphthylene-d10, phenanthrene-d10, chrysene-d12, and perylene-d12) and two nitro-PAH surrogates (9-nitroanthracene-d9 and 6-nitrochrysene-d11) were spiked onto the filters prior to extraction to monitor performance and matrix effects. The filters were then ultrasonically extracted with dichloromethane (DCM) for 30 min, which was repeated three times. The extracts were filtered, concentrated, and separated on a silica–alumina column. Two fractions were eluted: Fraction I (40 mL of hexane) including aliphatic hydrocarbons was discarded, while Fraction II (100 mL of 1:1 DCM-hexane), which contained the parent PAHs and nitro-PAHs, was collected and reduced to 5 ml, then dissolved in n-hexane. An internal standard, hexamethylbenzene, was added for the detection of parent PAHs and nitro-PAHs.

Extracted target compounds were quantified and analyzed by gas chromatography–mass spectrometry (GC-MS) using an Agilent 7890 N gas chromatograph coupled to an Agilent 5975 C inert mass selective detector operating in electron impact (EI) mode for parent-PAHs, and the electron capture negative ion (ECNI) and selected ion monitoring (SIM) modes for nitro-PAHs. The instrument was equipped with a HP-5MS (30 m × 0.25 mm × 0.25 μm) column to measure parent PAHs and most nitro-PAHs, and a DB–17MS column (60 m × 0.25 mm × 0.25 μm) was used to separate 2-nitrofluoranthene and 3-nitrofluoranthene. The oven temperature program for the analysis of parent PAHs was: 50 °C for 1 min, increased to 150 °C at a rate of 10 °C/min, to 240 °C at 3 °C/min, and then finally increased to 280 °C and held for 20 min. For nitro-PAHs, the oven temperature was increased to 150 °C from 60 °C at a rate of 15 °C/min, then to 300 °C at 5 °C/min and held at that temperature for 15 min. The carrier gas was high-purity helium. Target compounds were identified via qualitative analysis based on the retention time and standards.

### Quality control (QC) and quality assurance (QA)

Field blanks were performed every ten samples to quantify the background contamination. The concentrations of target compounds in the blanks were all under the instrument detection limits (IDLs) and therefore the results were not corrected for field blanks. Method detection limits (MDLs) were assigned as the average values of the field blanks plus three times the standard deviation of the field blank values. For compounds not found in the field blanks, the MDLs were calculated as three times the IDLs. In this study, MDLs for individual PAH compounds ranged from 18 to 200 pg/m^3^ and <3 to 25 pg/m^3^ for parent PAHs and nitro-PAHs, respectively. The mean recoveries of the parent PAH surrogates were 66 ± 12% for naphthalene-d8, 78 ± 10% for acenaphthene-d10, 99 ± 15% for phenanthrene-d10, 87 ± 5% for chrysene-d12, and 90 ± 16% for perylene-d12. The mean recoveries for the nitro-PAH surrogates were 79 ± 8% for 9-nitroanthracene-d9 and 88 ± 9% for 6-nitrochrysene-d11.

### PCA and PMF receptor model

PCA is a multivariate analytical tool used to reduce a set of original variables and extract a small number of latent principal components (PCs). PCA results can be used to explore relationships among the measured parameters and facilitate the assessment of possible factors. Before this analysis, values below the detection limit were replaced by one half of the method detection limit. In this study, the un-normalized concentrations of all PAHs in the PM2.5 samples were used for PCA (Table S2). SPSS (IBM SPSS Inc. statistics version) was used to extract the PCs from the correlation matrix. PCs with eigenvalues >1 were considered for further investigation.

The detailed background and applications of the PMF model for source apportionment were described in EPA PMF 5.0, Fundamentals and User Guide (http://www.epa.gov/heasd/research/pmf.html). In recent years, PMF has been successfully applied to spatially distributed datasets to determine the sources of parent PAHs in aerosol and sediment^24, 62–64^. In this study, the datasets used for PMF analysis included 26 species (14 parent PAHs and 12 nitro-PAHs, Table S2), which were input to the model using EPA PMF5.1 to determine the contributions of primary emissions and secondary formation sources of nitro-PAHs. The 5-factor solution was adopted in this study. Details of the analysis and quality control procedures used in this study are provided in supporting information S2.4.

### Health risk assessment

The toxicity equivalency (TEQ) methodology was developed by the US EPA to evaluate the toxicity and assess the risk of mixtures of structurally related chemicals with a common mechanism of action. A toxicity equivalency factor (TEF) is an estimate of the relative toxicity of a chemical compared to a reference chemical^65^. Because PAHs almost always occur as mixtures in the environment, their reference chemical is BaP. BaP was chosen as the reference chemical because its toxicity is well characterized^66^. Thus, the TEF for each parent PAH or nitro-PAH is an estimate of the relative toxicity of that compound compared to BaP in this study (Table S3), which has a TEF of 1. Then, the TEQ < BaP > for the mixture is calculated as:

1 $$TEQ < BaP > =\sum _{i=1}^{n}Ci\ast TEFi$$

where *C*i is the concentration of a compound in the mixture.

## Electronic supplementary material

Supplementary Information
