# Supplementary material for: Concentration, source identification, and exposure risk assessment of PM2.5-bound parent PAHs and nitro-PAHs in atmosphere from typical Chinese cities
Source: Sci Rep. 2017 Sep 4;7:10398. doi: 10.1038/s41598-017-10623-4 (PMC5583288; doi:10.1038/s41598-017-10623-4)
Supplement: Supplementary file 1 — Supplementary Information [file 41598_2017_10623_MOESM1_ESM.pdf]

Concentration, source identification, and exposure risk assessment of PM2.5-bound parent PAHs and nitro-PAHs in atmosphere from typical Chinese cities

Di Liu 1, Tian Lin 2*, Jabir Hussain Syed 1, Zhineng Cheng 1, Yue Xu 2, Kechang Li 1, Gan Zhang 1, Jun Li 1*

1 State Key Laboratory of Organic Geochemistry, Guangzhou Institute of Geochemistry, Chinese Academy of Sciences, Guangzhou 510640, China

2 State Key Laboratory of Environmental Geochemistry, Institute of Geochemistry, Chinese Academy of Sciences, Guiyang 550081, China

*Corresponding author: Tel/Fax: (86) 20 85290706;

Email: lintian@vip.gyig.ac.cn (T. Lin); junli@gig.ac.cn

Summary

S2.4 PMF receptor model	P3
Table S1. Details of sampling information	P5
Table S2. Concentrations of measured PAHs in PM2.5 from 9 urban cities	P10
Table S3. List of target compounds and TEF values	P18
Table S4. Particle-bound nitro-PAHs concentrations (pg/m3) at selected geographic locations.	P19
Figure S1. Percentage composition of 2-, 3-, 4-, 5- and 6-ring PAHs	P22
Figure S2. The relationships between the PMF predicted/modeled Q values for 4-factors, 5-factors and 6-factors results.	P23
Figure S3. 5-factor loadings by PMF analysis from parent PAHs and nitro-PAHs data of PM2.5 sample at nine urban sites across China.	P24
Figure S4. 4-factor loadings by PMF analysis from parent PAHs and nitro-PAHs data of PM2.5 sample at nine urban sites across China.	P26
Figure S5. 6-factor loadings by PMF analysis from parent PAHs and nitro-PAHs data of PM2.5 sample at nine urban sites across China.	P28

S2.4 PMF receptor model

Detailed concepts and applications of PMF model for source apportionment were described in EPA PMF 5.0 Fundamentals and User Guide (http://www.epa.gov/heasd/research/pmf.html). In principle, the PMF model is based on the following equations:

$$Xij = \sum_{k=1}^{p} Aik\ Fkj + Rij \quad \text{E.g.1}$$

where Xij is the concentration of the jth congener in the ith sample of the original data sets; Aik is the contribution of the kth factor to the ith sample; Fkj is the fraction of the kth factor arising from congener j; Rij is the residual between the measured Xij and the predicted Xij using p principal components.

$$Q = \sum_{i=1}^{n} \sum_{j=1}^{m} \left(\frac{Xij - \sum_{k=1}^{p} Aik\ Fkj}{Sij}\right)^2 \quad \text{E.g.2}$$

where Sij is the uncertainty of the jth congener in the ith sample of the original data sets containing m congeners and n samples. Q is the weighted sum of squares of differences between the PMF output and the original data sets. One of the objectives of PMF analysis is to minimize the Q value. Before the PMF analysis, both the concentration file and uncertainty file were inserted into the model. In this study, uncertainties of 20% for parent PAHs and 15% for nitro-PAHs were adopted based on the results from QA/QC, respectively.

During the PMF analysis, the model was run for 3—7 factors and was always with random seeds. Finally, the five-factor solution was adopted for further discussion in this study. The 5-factor results adopted in this study were based on: 1) The correlation between the model estimated concentrations and the measured concentrations was highest for 5-factor result (See Figure S2). Besides, Q is the weighted sum of squares of the differences between the PMF output and the measured concentration, and PMF aims to minimize the Q value. This solution produced Q values

(both robust and true) close to the theoretical Q value (deviation <4%), indicating the appropriate uncertainties provided for PAHs data sets in the modeling input. Although lower Q values could be obtained when adding more factors (6 or more), more factors resulted in no substantial improvement in the ability to interpret the factor profiles (See the figure S3-5). Thus, 5-factor solution was adopted which gave the most stable results and the easily interpretable sources by comparing to the source profiles from acknowledged references in China. (2) The potential PAH sources also provide important support to the 5-factor results. According to the PAH emission inventory of China, biomass burning (>40%), coal combustion and vehicular emission contributes over 90% of total PAH emission (Xu et al., 2006). Residential emissions were significant contributed to PAHs in the urban area. Secondary formation was important source of nitro-PAHs in the urban area.

Even without consideration the Q value, for the PMF analysis the 4-factor results merely divided the PAHs into nitro-PAHs, Nap, low molecular weight PAHs and high molecular. For 6-factor results, it unable to give the meaningful identification in the light of the possible PAH sources according to the source profiles comparing with previous studies.

Table S1. Details of sampling information.

Cites	Abbr.	Coordinates	Descriptions
Beijing	B	39.93N, 116.34E	The sampling site was selected on five-floor rooftop (15 m height) of the building in National Research Center for Geoanalysis, located at the central district surrounded by plenty of schools and institutes.
Shanghai	S	31.29N, 121.50E	The sampling site was selected on five-floor rooftop of the building in the Handan Campus of Fudan University, surrounded by dwellings and commercial buildings.
Guangzhou	G	23.15N, 113.36E	The sampling site was selected on five-floor rooftop of the building in Institute of Geochemistry, CAS, which is close to the trunk road and train station, surrounded by dwellings and schools.
Nanjing	N	32.06N, 118.80E	The sampling site was selected on five-floor rooftop of the building (Institute of Soil Science, CAS) in the district by a large population density.
Chengdu	C	30.64N, 104.08E	The sampling site was selected on rooftop of the building in Institute of Mountain Hazards and Environment, CAS, which is surrounded by dwellings.
Wuhan	W	30.53N, 114.37E	The sampling site was selected on five-floor rooftop of the building in Wuhan University, which is surrounded by parks, dwellings and schools, far from the trunk road.
Xinxiang	X	35.33N, 113.91E	The sampling site was selected on five-floor rooftop (15 m height) of the building in Henan Normal University, surrounded by plenty of factories.
Lanzhou	L	36.05N, 103.86E	The sampling site was selected on eight-floor rooftop of the building in Lanzhou University, which is surrounded by mountains on the north and south.
Taiyuan	T	37.54N, 112.33E	The sampling site is selected on nine-floor rooftop of the building in China Institute for Radiation Protection, surround by dwellings and commercial buildings

Annex to Table S1.

Season	City	No.	Sampling date
**Autumn**	Beijing	B31	2013/10/22
		B32	2013/10/29
		B33	2013/11/07
		B34	2013/11/12
	Chengdu	C31	2013/10/22
		C32	2013/10/29
		C33	2013/11/05
		C34	2013/11/12
	Lanzhou	L31	2013/10/23
		L32	2013/10/29
		L33	2013/11/05
		L34	2013/11/12
	Wuhan	W31	2013/10/17
		W32	2013/10/22
		W33	2013/10/29
		W34	2013/11/05
	Taiyuan	T31	2013/10/22
		T32	2013/10/29
		T33	2013/11/05
		T34	2013/11/12
	Xinxiang	X31	2013/10/22
		X32	2013/10/29
		X33	2013/11/05
		X34	2013/11/12
	Guangzhou	G31	2013/10/22
		G32	2013/10/30
		G33	2013/11/05
		G34	2013/11/12
	Nanjing	N31	2013/10/20
		N32	2013/10/28
		N33	2013/11/05
		N34	2013/11/13
	Shanghai	S31	2013/10/22
		S32	2013/10/30
		S33	2013/11/05
		S34	2013/11/13

(Continued)

Season	City	No.	Sampling date
**Winter**	Beijing	B41	2013/12/30
		B42	2014/01/06
		B43	2014/01/13
		B44	2014/01/20
	Chengdu	C41	2013/12/30
		C42	2014/01/06
		C43	2014/01/13
		C44	2014/01/20
	Lanzhou	L41	2013/12/30
		L42	2014/01/06
		L43	2014/01/13
		L44	2014/01/20
	Wuhan	W41	2013/12/30
		W42	2014/01/02
		W43	2014/01/11
		W44	2014/01/20
	Taiyuan	T41	2013/12/30
		T42	2014/01/06
		T43	2014/01/13
		T44	2014/01/20
	Xinxiang	X41	2013/12/30
		X42	2014/01/06
		X43	2014/01/13
		X44	2014/01/20
	Guangzhou	G41	2013/12/31
		G42	2014/01/06
		G43	2014/01/13
		G44	2014/01/19
	Nanjing	N41	2013/12/31
		N42	2014/01/06
		N43	2014/01/14
		N44	2014/01/19
	Shanghai	S41	2013/12/30
		S42	2014/01/06
		S43	2014/01/13
		S44	2014/01/20

(Continued)

Season	City	No.	Sampling date
**Spring**	Beijing	B11	2014/04/13
		B12	2014/04/20
		B13	2014/04/27
		B14	2014/05/04
	Chengdu	C11	2014/03/30
		C12	2014/04/06
		C13	2014/04/13
		C14	2014/04/20
	Lanzhou	L11	2014/03/30
		L12	2014/04/06
		L13	2014/04/13
		L14	2014/04/20
	Wuhan	W11	2014/04/06
		W12	2014/04/13
		W13	2014/04/21
		W14	2014/04/27
	Taiyuan	T11	2014/03/30
		T12	2014/04/06
		T13	2014/04/13
		T14	2014/04/20
	Xinxiang	X11	2014/03/29
		X12	2014/04/06
		X13	2014/04/13
		X14	2014/04/20
	Guangzhou	G11	2014/03/30
		G12	2014/04/06
		G13	2014/04/13
		G14	2014/04/20
	Nanjing	N11	2014/03/28
		N12	2014/04/05
		N13	2014/04/14
		N14	2014/04/19
	Shanghai	S11	2014/03/23
		S12	2014/03/30
		S13	2014/04/06
		S14	2014/04/13

(Continued)

Season	City	No.	Sampling date
**Summer**	Beijing	B21	2014/08/03
		B22	2014/08/10
		B23	2014/08/17
		B24	2014/08/24
	Chengdu	C21	2014/06/28
		C22	2014/07/05
		C23	2014/07/12
		C24	2014/07/18
	Lanzhou	L21	2014/06/28
		L22	2014/07/05
		L23	2014/07/12
		L24	2014/07/19
	Wuhan	W21	2014/07/12
		W22	2014/07/19
		W23	2014/07/23
		W24	2014/07/26
	Taiyuan	T21	2014/06/23
		T22	2014/06/28
		T23	2014/07/05
		T24	2014/07/12
	Xinxiang	X21	2014/07/19
		X22	2014/07/24
		X23	2014/07/29
		X24	2014/08/01
	Guangzhou	G21	2014/06/22
		G22	2014/06/28
		G23	2014/07/05
		G24	2014/07/12
	Nanjing	N21	2014/06/28
		N22	2014/07/04
		N23	2014/07/12
		N24	2014/07/19
	Shanghai	S21	2014/06/28
		S22	2014/07/05
		S23	2014/07/12
		S24	2014/07/20

Table S2/1. Concentrations of parent PAHs in PM2.5 from the atmosphere of Chinese cites (ng/m3).

	Nap	Fl	Phe	Ant	Flu	Pyr	BaA	CHr	BbF	BkF	BaP	IP	DBA	BghiP
Beijing														
B11	0.32	0.53	1.53	0.27	5.12	4.58	4.18	5.50	8.41	1.71	3.37	4.62	0.13	3.55
B12	0.28	0.39	2.51	0.08	3.46	3.19	2.62	2.72	6.47	1.89	2.52	3.08	0.31	2.57
B13	0.18	0.25	0.91	0.06	1.50	1.46	1.06	0.91	3.07	0.73	1.17	1.77	0.11	1.37
B14	0.49	0.48	1.06	0.24	1.75	1.02	0.85	1.04	1.14	0.44	0.69	0.49	0.13	0.11
B21	0.12	0.14	0.23	0.02	0.41	0.33	0.43	0.53	0.90	0.33	0.52	0.43	0.11	0.39
B22	0.29	0.15	0.27	0.01	0.43	0.32	0.44	0.52	0.99	0.16	0.52	0.44	0.11	0.49
B23	0.02	0.14	0.19	0.03	0.43	0.40	0.57	0.59	0.99	0.47	0.33	0.44	0.10	0.44
B24	0.10	0.14	0.23	0.01	0.52	0.48	0.58	0.67	0.99	0.42	0.58	0.42	0.10	0.28
B31	0.19	0.33	1.00	0.20	4.37	4.47	6.27	9.71	10.63	4.10	5.70	0.62	0.16	0.14
B32	0.22	0.32	1.34	0.25	4.27	3.56	4.40	4.36	5.14	0.66	1.01	0.47	0.12	0.11
B33	0.25	0.14	0.48	0.20	1.55	1.25	1.36	1.94	1.72	0.36	0.93	0.44	0.11	0.10
B34	0.01	0.15	2.37	0.15	10.16	10.08	8.76	12.47	11.95	2.61	5.78	0.45	0.11	0.11
B41	0.00	0.23	2.75	0.27	7.05	5.00	2.76	2.68	4.36	0.86	1.56	2.03	0.28	1.38
B42	0.95	1.19	20.78	2.98	68.07	66.81	37.19	96.40	60.52	10.11	32.22	27.07	3.57	28.04
B43	0.02	0.23	3.35	0.49	11.55	10.08	12.23	14.60	16.97	3.42	6.82	6.25	0.55	4.71
B44	0.03	0.24	6.75	1.18	32.14	32.94	29.34	50.98	2.39	23.41	21.89	15.03	1.66	11.55
Chengdu														
C11	0.03	0.15	0.59	0.13	1.88	1.97	2.11	1.89	7.03	1.32	2.52	4.45	0.33	3.63
C12	0.07	0.15	0.35	0.11	1.40	1.34	1.21	1.07	4.32	0.95	1.49	2.86	0.23	2.35
C13	0.11	0.14	0.25	0.08	0.72	0.66	1.29	0.70	2.57	0.81	1.56	1.82	0.21	1.36
C14	0.16	0.14	0.32	0.07	0.60	0.62	0.44	0.11	0.26	0.15	0.34	0.43	0.10	0.10
C21	0.01	0.13	0.21	0.01	0.42	0.38	0.44	0.44	1.06	0.35	0.56	0.40	0.10	0.47
C22	0.03	0.15	0.02	0.02	0.46	0.44	0.65	0.67	0.28	0.28	0.38	0.45	0.11	0.10
C23	0.14	0.14	0.24	0.07	0.56	0.45	0.80	0.71	2.26	0.37	0.84	1.21	0.11	0.91
C31	nd	0.16	0.69	0.08	1.86	1.95	2.09	2.43	5.13	1.35	2.07	0.46	0.11	0.24
C32	0.05	0.14	0.75	0.11	2.50	2.47	1.90	2.74	4.85	1.03	1.65	0.44	0.11	0.64
C33	0.04	0.15	0.41	0.04	1.39	1.42	1.67	1.55	4.16	1.07	1.25	0.44	0.10	0.25
C34	0.05	0.14	0.27	0.03	0.85	0.81	0.72	0.62	1.41	0.57	0.76	0.42	0.10	0.10
C41	0.06	0.19	1.15	2.02	5.38	4.86	4.38	4.26	10.46	2.03	4.77	2.42	0.34	2.46
C42	0.03	0.14	1.44	0.06	5.19	4.96	4.92	6.66	8.74	1.40	4.82	3.46	0.29	2.77
C43	nd	0.15	0.85	0.07	2.90	2.63	4.94	4.55	2.82	1.19	1.50	1.86	0.20	1.30
C44	0.08	0.15	0.78	0.03	3.03	3.02	2.65	3.54	5.61	1.18	2.40	2.76	0.31	2.08
Guangzhou														
G11	0.80	0.20	0.67	0.16	0.97	1.16	1.25	3.18	5.57	1.82	2.15	3.34	0.35	3.35
G12	0.04	0.20	0.53	0.03	0.72	0.71	0.71	0.77	2.09	0.72	0.99	0.63	0.16	0.14
G13	0.08	0.19	0.60	0.08	0.50	0.57	0.63	0.69	3.01	0.64	0.97	2.53	0.20	1.96
G14	0.04	0.23	0.54	0.06	0.51	0.52	0.56	0.55	2.39	0.84	0.84	2.06	0.14	1.60
G21	0.19	0.18	0.39	0.03	0.49	0.49	0.74	0.48	1.80	0.60	0.85	0.53	0.13	0.12
G22	0.20	0.18	0.35	0.12	0.41	0.28	0.56	0.18	0.38	0.18	0.42	0.54	0.13	0.12
G23	0.03	0.18	0.38	0.07	0.50	0.60	0.60	0.47	2.89	0.69	1.52	1.41	0.12	1.16

G24	0.21	0.18	0.26	0.11	0.33	0.31	0.43	0.37	1.27	0.23	0.74	1.10	0.13	1.19
G31	0.37	0.42	0.86	0.25	1.29	1.37	1.85	1.82	5.81	1.00	2.64	1.74	0.15	1.57
G32	0.06	0.18	0.44	0.03	0.91	0.98	0.82	0.99	1.95	0.87	0.86	1.76	0.13	1.03
G33	nd	0.12	0.12	0.02	0.28	0.37	0.32	0.26	0.42	0.23	0.32	0.38	0.09	0.09
G41	0.26	0.41	3.61	0.33	7.11	6.18	5.30	7.73	12.61	2.59	5.59	7.30	0.55	5.93
G42	0.07	0.23	1.12	0.07	2.09	1.86	2.06	2.37	3.84	1.13	1.72	0.61	0.15	0.45
G43	nd	0.17	0.70	0.08	1.76	1.97	0.91	1.26	3.21	0.93	1.33	1.85	0.13	1.05
G44	0.11	0.28	2.21	0.09	4.77	4.41	2.09	2.22	1.74	0.66	0.77	0.56	0.14	0.13
Lanzhou														
L11	0.32	0.14	1.28	0.22	4.11	3.89	1.96	2.78	4.41	1.65	1.13	0.43	0.10	0.10
L12	0.15	0.14	0.49	0.08	1.97	1.90	1.20	2.06	2.50	0.55	0.93	0.44	0.11	0.68
L13	0.02	0.20	0.99	0.21	3.38	3.32	3.63	3.45	7.81	2.15	2.79	3.18	0.49	2.62
L14	0.07	0.16	0.41	0.08	1.53	1.71	1.41	1.34	3.61	0.61	1.29	1.83	0.12	1.56
L21	0.07	0.15	0.24	0.02	0.40	0.25	0.52	0.57	1.28	0.46	0.40	0.48	0.11	0.19
L22	0.07	0.16	0.60	0.09	1.57	1.49	1.47	2.01	3.33	0.99	1.30	1.54	0.24	1.12
L23	0.23	0.16	0.93	0.04	2.02	1.96	1.91	2.53	4.23	1.10	1.59	0.48	0.11	0.37
L24	0.20	0.29	0.22	0.27	0.55	0.51	1.51	2.51	1.23	1.14	1.27	1.22	0.64	0.60
L31	0.07	0.14	1.36	0.12	6.18	6.40	4.48	4.84	5.28	1.36	2.22	0.44	0.11	0.10
L32	0.09	0.15	1.25	0.20	7.46	8.50	8.32	10.66	14.23	2.62	6.78	2.46	0.27	2.41
L33	0.23	0.31	0.87	0.26	7.20	7.20	6.24	9.40	12.93	2.53	3.37	4.41	0.57	3.65
L41	0.04	1.59	32.52	4.78	82.65	74.41	35.16	33.49	55.58	38.59	0.43	21.34	2.35	16.38
L42	0.39	1.88	42.13	5.40	97.78	91.02	43.45	89.06	76.98	54.97	32.32	24.95	2.83	20.06
L43	0.43	0.44	50.01	11.33	122.47	106.01	19.36	30.47	61.98	20.68	44.27	0.96	0.35	23.43
L44	0.43	0.74	11.61	2.76	41.49	38.99	12.92	18.20	25.07	4.39	11.57	13.99	2.07	11.01
Nanjing														
N11	0.09	0.13	0.19	0.04	0.40	0.39	0.48	0.38	0.98	0.32	0.46	0.40	0.10	0.32
N12	0.02	0.21	1.54	0.16	2.97	2.22	2.20	3.25	4.67	0.88	2.03	2.20	0.19	1.71
N13	0.13	0.12	0.24	0.10	0.56	0.59	0.61	0.71	1.54	0.67	0.77	0.90	0.09	0.85
N14	0.00	0.14	0.10	0.01	0.26	0.18	0.26	0.26	0.62	0.28	0.34	0.43	0.10	0.10
N21	0.08	0.14	0.21	0.07	0.84	0.64	0.64	0.55	1.38	0.78	0.94	0.43	0.11	0.64
N22	0.00	0.13	0.09	0.02	0.27	0.24	0.39	0.35	0.25	0.30	0.31	0.40	0.10	0.09
N23	0.18	0.13	0.22	0.24	0.49	0.43	1.00	1.12	2.38	1.01	1.33	0.38	0.10	0.45
N24	0.07	0.13	0.24	0.06	0.39	0.32	1.10	1.02	1.03	0.70	0.78	0.96	0.49	0.56
N31	nd	0.14	0.49	0.61	1.11	1.12	0.40	0.45	2.46	0.22	0.89	0.79	0.11	0.44
N32	0.20	0.33	0.79	0.22	1.43	1.45	1.59	2.22	3.10	0.81	1.28	2.29	0.21	1.64
N33	0.19	0.13	0.59	0.05	1.52	1.53	1.66	2.21	3.83	0.69	1.50	1.66	0.09	1.17
N41	0.09	0.17	3.93	0.20	9.20	8.61	4.72	6.51	8.34	1.77	3.12	4.01	0.37	4.08
N42	0.08	0.33	1.61	0.17	3.96	3.62	3.82	4.75	7.74	1.24	2.37	4.04	0.30	3.38
N43	0.11	0.25	1.36	0.21	3.06	2.26	2.03	3.07	4.11	1.00	1.68	2.26	0.20	1.45
N44	0.20	0.45	4.39	5.54	10.30	8.28	4.33	6.39	8.18	1.59	3.29	1.86	0.49	2.09
Shanghai														
S11	0.10	0.13	0.44	0.07	0.92	0.72	0.68	0.76	1.14	0.35	0.54	0.85	0.09	0.47
S12	nd	0.14	0.22	0.27	0.81	0.72	0.30	0.34	2.13	0.36	0.60	0.74	0.10	0.40

S13	0.34	0.15	0.34	0.06	0.69	0.52	0.44	0.64	1.28	0.38	0.51	0.43	0.10	0.23
S14	0.04	0.14	0.12	0.02	0.41	0.36	0.37	0.46	1.57	0.51	0.48	0.43	0.10	0.48
S21	0.08	0.14	0.21	0.02	0.32	0.29	0.28	0.19	0.27	0.15	0.36	0.43	0.10	0.10
S22	nd	0.14	0.12	0.01	0.26	0.12	0.47	0.19	0.27	0.16	0.33	0.42	0.10	0.10
S23	nd	0.15	0.13	0.02	0.24	0.16	0.26	0.18	0.64	0.31	0.36	0.44	0.11	0.10
S31	0.03	0.12	0.10	0.03	0.23	0.16	0.33	0.29	0.89	0.20	0.29	0.36	0.09	0.36
S32	0.01	0.13	0.36	0.06	0.64	0.54	0.87	1.11	2.22	0.78	0.98	1.68	0.13	1.06
S33	nd	0.17	0.07	0.07	0.88	1.07	0.22	0.28	0.32	0.19	0.42	0.58	0.14	0.12
S41	0.19	0.40	9.36	0.56	18.23	14.61	8.43	11.34	15.65	3.09	7.05	7.95	0.59	6.52
S42	nd	0.12	1.46	1.84	2.90	2.12	1.37	1.38	2.67	0.20	0.49	0.38	0.09	0.09
Taiyuan														
T11	0.72	0.74	2.96	0.43	9.40	7.43	5.10	6.53	9.22	1.83	3.91	2.48	0.13	2.55
T12	0.17	0.40	5.96	0.53	13.96	10.68	7.92	10.54	13.13	2.41	5.13	3.12	0.53	2.98
T13	1.03	0.73	6.26	0.60	20.04	15.39	16.66	47.47	32.31	6.19	9.81	9.61	1.44	10.08
T21	0.20	0.26	1.04	0.09	2.68	2.18	1.86	1.91	4.93	1.23	2.03	2.39	0.33	1.79
T22	0.29	0.41	0.93	0.23	2.65	2.12	1.78	2.45	7.29	1.14	1.64	3.02	0.86	2.25
T23	0.24	0.40	0.81	0.23	2.67	1.74	2.21	2.62	2.94	0.63	1.14	0.46	0.12	0.11
T24	0.18	0.16	0.69	0.06	2.02	1.67	1.10	1.34	2.45	0.74	1.11	1.30	0.11	0.86
T31	0.37	0.44	7.05	9.71	32.29	24.42	32.89	51.58	48.76	7.20	12.36	11.57	1.24	10.77
T32	0.86	1.41	15.56	1.57	60.37	51.32	51.23	89.43	93.14	66.39	43.48	34.56	3.37	34.44
T33	0.12	0.45	14.25	1.13	49.11	41.59	35.43	52.99	62.89	12.73	25.97	24.99	3.17	23.01
T34	nd	0.17	9.52	12.02	25.49	20.82	11.93	15.16	20.60	3.82	9.23	6.30	0.76	6.77
T41	0.16	2.08	56.25	6.23	103.13	79.63	32.15	33.09	58.82	42.06	29.81	22.71	3.46	24.20
T42	0.02	1.09	37.80	6.03	95.26	82.30	36.49	45.45	64.07	14.55	36.33	28.29	3.08	25.80
T43	0.02	2.77	62.71	10.84	120.50	93.61	32.76	33.56	58.34	8.11	31.71	21.49	2.94	21.75
T44	0.05	0.68	6.30	0.83	16.03	11.72	4.39	4.30	7.25	1.54	2.92	3.17	0.58	2.81
Wuhan														
W11	0.14	0.11	0.46	0.05	2.72	2.32	1.83	1.71	4.12	0.88	1.68	1.76	0.29	1.62
W12	0.03	0.09	0.12	0.01	0.69	0.58	0.75	0.76	2.10	0.52	0.46	0.27	0.07	0.30
W13	nd	0.09	0.26	0.04	1.51	1.05	1.10	0.99	1.58	0.40	0.39	0.27	0.06	0.06
W14	0.13	0.09	0.43	0.02	0.79	0.63	0.50	1.14	1.70	0.62	0.40	0.63	0.07	0.34
W21	0.06	0.08	0.25	0.01	0.58	0.47	0.35	0.41	1.41	0.40	0.74	1.17	0.12	0.79
W22	0.16	0.09	0.38	0.09	0.92	0.88	0.66	0.85	2.15	0.49	1.04	0.90	0.15	0.80
W23	nd	0.09	0.30	0.37	0.51	0.39	0.16	0.16	1.40	0.14	0.51	0.28	0.07	0.29
W31	nd	0.13	0.97	0.07	3.92	3.33	2.19	2.59	4.97	1.11	1.68	1.65	0.16	1.36
W32	0.03	0.10	1.14	0.09	4.02	3.14	2.42	3.17	1.60	1.13	1.59	0.13	0.03	0.34
W33	0.06	0.08	0.65	0.08	1.59	1.36	1.72	1.98	3.30	0.59	0.79	0.26	0.06	0.32
W34	0.03	0.08	0.53	0.03	1.73	1.51	3.06	3.91	8.71	1.43	1.67	3.64	0.34	3.33
W41	0.02	0.13	1.30	0.17	3.55	3.19	2.48	2.63	5.13	0.81	2.41	1.09	0.11	1.05
W42	nd	0.10	1.45	0.15	4.45	3.76	7.98	10.12	3.84	4.07	8.12	3.71	0.33	3.04
W43	0.60	1.02	4.95	2.19	11.26	9.37	5.68	13.84	15.07	2.12	2.72	0.29	0.11	3.86
Xinxiang														
X11	nd	0.14	1.05	1.32	3.70	3.29	3.01	3.74	7.17	1.64	1.84	2.72	0.12	2.36

X12	nd	0.15	0.68	0.85	2.84	2.50	2.61	2.99	7.38	1.75	2.20	0.97	0.12	1.08
X13	0.14	0.14	0.38	0.10	0.89	0.81	1.24	0.78	2.81	0.64	1.17	0.45	0.11	0.30
X21	0.27	0.16	0.65	0.16	1.56	1.50	1.13	1.45	3.67	0.83	1.78	1.53	0.25	1.35
X22	0.03	0.38	0.88	0.76	3.44	2.95	2.34	3.18	6.66	4.73	1.94	0.57	0.11	0.26
X23	nd	0.16	0.53	0.72	0.90	1.15	0.98	1.06	3.47	2.31	2.33	1.09	0.11	0.97
X31	0.18	0.42	2.37	0.43	14.18	15.68	16.16	32.72	28.75	6.49	14.55	7.32	1.10	7.95
X32	0.10	0.24	1.68	0.09	7.50	6.54	7.52	8.43	17.59	3.84	6.19	5.97	0.42	5.95
X41	0.49	0.72	10.67	13.47	31.98	25.55	22.53	40.55	28.26	20.48	8.05	8.19	0.88	7.57
X42	nd	0.40	11.10	1.07	32.26	25.29	23.57	29.13	5.11	22.88	9.93	12.20	0.99	9.34
X43	0.06	0.22	7.89	0.78	36.93	32.33	19.56	21.79	0.42	19.62	0.52	13.26	1.25	10.82
X44	0.44	0.74	3.61	0.56	12.21	9.79	6.59	8.13	9.54	1.89	3.49	4.42	0.28	3.99

Table S2/2. Concentrations of nitro-PAHs in PM2.5 from the atmosphere of Chinese cites (pg/m3).

	1n-Nap	2n-Nap	5n-Ace	2n-Fl	9n-Phe	3n-Phe	9n-Ant	2n-Ant	3n-Flu	1n-Pyr	7n-BaA	6n-Chr
Beijng												
B11	101.7	252.0	34.6	2.9	6.0	23.6	627.3	87.3	46.5	34.6	54.1	3.2
B12	99.7	225.0	18.4	1.0	2.1	20.3	458.7	235.1	29.0	26.7	28.9	3.0
B13	99.0	222.5	18.0	1.0	2.0	19.9	158.5	144.6	29.2	26.7	28.1	2.9
B14	109.0	244.6	20.3	1.2	2.3	22.3	171.5	216.2	32.3	29.3	30.4	3.5
B21	98.4	220.4	17.9	1.0	13.5	19.5	151.9	43.6	28.4	26.2	27.5	2.8
B22	100.2	226.7	18.4	1.1	8.0	20.3	155.6	48.9	29.8	29.1	28.8	3.0
B23	98.0	220.3	17.9	1.1	9.0	19.4	151.8	19.7	28.4	25.9	27.4	2.9
B24	96.0	215.2	17.5	1.0	7.5	19.0	150.3	19.1	27.6	25.6	26.9	2.8
B31	141.4	308.3	25.3	4.9	11.4	32.2	713.5	55.3	42.9	42.1	40.8	4.3
B32	106.6	239.2	19.5	1.1	9.5	21.5	197.1	23.7	31.5	29.1	30.7	3.2
B33	98.1	220.3	18.0	2.3	2.0	20.4	158.7	20.5	29.0	26.4	27.8	2.9
B34	102.3	228.8	19.0	1.1	3.1	21.3	853.7	26.4	31.2	27.6	29.1	3.1
B41	97.3	219.2	18.2	3.8	2.0	20.3	191.1	22.1	28.4	26.6	27.1	2.8
B42	103.3	225.2	21.2	1.8	3.1	33.7	5981.9	26.1	33.8	31.7	33.3	3.3
B43	108.8	232.4	19.2	5.8	3.0	21.6	1038.6	343.0	31.0	29.6	31.3	3.2
B44	97.5	217.3	17.8	12.0	7.3	34.6	2106.3	64.2	162.7	33.3	28.3	2.9
Chengdu												
C11	105.0	237.7	19.2	1.0	2.1	21.2	388.5	21.5	31.4	28.7	29.2	3.1
C12	104.4	230.3	18.8	1.0	2.1	20.4	245.4	20.6	29.5	27.3	28.4	3.0
C13	100.4	222.4	18.1	1.0	4.2	19.6	181.7	19.9	28.6	26.1	27.9	2.9
C14	96.7	215.1	17.5	1.0	1.9	19.1	148.6	19.0	27.8	25.5	26.9	2.8
C21	90.5	202.7	16.4	0.9	1.8	18.2	140.2	18.3	26.2	23.9	25.6	2.6
C22	100.5	226.5	18.5	1.0	2.0	20.5	160.4	20.2	29.2	26.9	28.2	2.9
C23	97.8	219.7	17.9	1.0	4.9	19.6	159.4	19.3	28.2	25.9	27.3	2.8
C31	104.9	235.8	19.2	1.1	2.2	21.3	180.3	22.2	31.9	27.9	29.6	3.1
C32	99.6	220.8	18.1	1.7	2.0	20.1	154.6	20.6	28.7	26.7	28.3	2.9
C33	98.6	221.8	18.2	1.0	2.0	19.7	154.0	20.1	29.3	26.2	27.4	2.9
C34	95.9	213.9	17.5	1.0	1.9	19.4	147.8	19.2	27.8	25.6	27.2	2.8
C41	98.6	222.8	18.3	3.5	2.7	20.3	203.1	20.7	29.2	29.1	28.4	2.9
C42	96.1	218.1	19.2	1.1	1.9	19.9	550.3	20.5	28.4	26.2	27.7	2.9
C43	102.6	229.8	18.9	1.0	2.1	20.9	481.4	236.6	31.4	28.8	29.6	3.0
C44	100.3	222.9	43.0	1.0	2.0	20.9	405.3	20.1	29.7	26.8	29.1	2.9
Guangzhou												
G11	139.0	303.9	24.9	1.4	8.5	27.2	2124.5	27.5	40.0	37.0	38.3	4.0
G12	142.1	320.6	25.7	1.4	2.9	28.2	2169.8	28.2	41.2	37.8	39.8	4.1
G13	130.2	291.0	23.7	1.3	12.6	26.0	2013.5	26.2	37.8	34.4	36.2	3.8
G14	118.9	508.9	21.7	1.2	32.8	24.0	1992.6	24.8	34.3	88.4	33.7	3.4
G21	119.8	270.4	21.9	1.2	10.9	24.2	857.5	24.5	35.0	32.2	33.4	3.5
G22	121.8	273.7	22.3	1.3	17.4	24.3	736.7	24.2	35.1	32.2	33.8	3.5
G23	117.8	266.4	21.6	1.2	10.0	23.6	1002.6	24.4	34.3	31.5	33.3	3.4

G24	121.8	272.4	22.2	1.2	21.3	24.3	653.2	24.4	35.3	32.1	34.4	3.5
G31	137.7	309.6	24.9	1.9	5.2	27.5	290.1	28.6	47.2	45.5	41.2	4.3
G32	125.6	282.5	23.0	1.2	4.3	25.1	194.9	25.0	36.6	33.4	35.4	3.6
G33	85.7	193.2	15.7	0.9	5.1	17.3	366.6	17.1	25.3	22.8	24.2	2.5
G41	126.8	284.7	23.4	1.4	3.0	25.8	1101.6	26.5	37.3	34.0	35.8	3.8
G42	138.1	309.2	25.1	1.4	2.8	27.7	215.9	27.8	39.9	37.1	38.3	4.1
G43	119.6	271.0	21.9	1.2	2.4	24.1	208.8	23.7	34.9	31.9	33.5	3.5
G44	128.3	290.0	23.3	2.0	5.8	26.0	198.8	25.7	37.0	33.7	36.0	3.7
Lanzhou												
L11	96.6	215.1	18.0	1.0	11.5	20.4	150.4	126.6	28.6	25.5	30.8	2.9
L12	97.5	219.8	18.0	1.0	3.6	19.6	260.2	19.9	29.5	26.1	27.8	2.9
L13	125.9	281.9	23.0	3.5	2.5	25.7	197.8	25.9	36.6	33.7	35.4	3.7
L14	106.6	236.2	19.3	1.1	2.1	21.7	168.5	21.6	30.9	28.5	30.0	3.1
L21	107.4	241.5	19.6	1.1	4.6	21.5	166.6	21.4	31.4	28.5	30.5	3.1
L22	102.3	228.3	18.6	1.0	7.1	21.0	159.2	21.0	29.6	27.9	28.3	3.0
L23	107.4	235.4	19.5	1.0	2.2	21.2	233.2	21.4	30.8	28.1	30.2	3.1
L24	100.2	226.6	18.5	1.0	7.4	20.5	157.0	20.2	29.5	27.2	29.8	2.9
L31	99.8	224.1	18.2	4.7	2.1	20.9	240.5	21.1	30.6	26.8	28.7	3.2
L32	103.0	227.0	18.8	6.6	2.2	22.8	686.8	20.9	31.6	28.1	30.1	3.0
L33	100.8	225.3	18.5	1.1	2.0	22.7	861.8	21.2	29.2	27.0	33.2	2.9
L41	100.6	225.8	21.9	35.0	7.9	30.3	855.6	205.0	37.0	28.2	35.6	3.2
L42	99.6	222.7	31.1	32.4	7.2	48.6	449.1	20.6	126.5	67.1	28.5	3.0
L43	398.1	438.7	55.8	2.8	10.7	94.1	810.3	163.4	97.8	109.6	83.6	10.5
L44	94.9	215.0	18.8	11.7	2.4	32.2	1386.0	23.5	40.7	28.2	26.5	3.0
Nanjing												
N11	90.2	204.0	16.5	0.9	10.8	17.9	146.8	18.2	26.3	24.0	25.1	2.6
N12	88.9	198.2	16.1	1.2	6.6	17.9	186.9	17.7	25.8	23.4	25.4	2.6
N13	87.0	195.0	15.9	0.9	6.0	17.5	139.1	17.4	25.3	23.2	24.4	2.5
N14	97.2	218.4	17.7	1.0	5.2	19.3	150.9	19.3	28.0	25.7	26.9	2.8
N21	96.1	217.0	17.5	1.0	10.4	19.3	170.5	20.0	27.8	25.5	27.5	2.8
N22	90.7	203.3	16.6	0.9	7.2	18.0	146.5	18.0	26.7	24.2	25.3	2.6
N23	87.8	198.7	15.9	0.9	19.1	17.7	137.8	18.1	25.3	23.1	24.2	2.5
N24	89.5	200.5	16.3	0.9	12.1	17.8	147.2	17.7	26.0	23.7	25.1	2.6
N31	95.8	216.0	17.6	1.0	1.9	19.2	150.4	19.7	28.5	25.6	27.3	2.8
N32	100.1	223.0	18.3	1.0	2.0	20.3	154.8	20.0	29.1	27.3	28.0	2.9
N33	86.4	192.2	15.7	0.8	1.8	17.4	219.0	17.5	25.7	23.1	24.3	2.6
N41	83.3	185.9	18.5	3.7	1.7	18.2	701.2	17.1	24.7	22.4	24.2	2.6
N42	96.3	216.3	17.5	1.0	2.1	19.6	625.3	20.5	29.0	26.2	27.2	2.9
N43	86.9	195.5	16.1	2.0	1.8	17.7	199.1	17.5	25.4	23.2	25.0	2.5
N44	91.8	205.4	16.9	2.5	1.9	19.1	433.6	18.2	26.9	24.4	25.8	2.7
Shanghai												
S11	87.2	195.5	15.9	0.9	3.4	17.7	135.5	17.6	25.6	23.0	24.1	2.5

S12	99.0	219.1	18.0	1.1	3.7	19.6	156.6	19.3	28.5	25.9	27.7	2.8
S13	97.0	218.2	17.8	1.0	2.0	19.6	149.6	19.4	28.2	25.9	27.0	2.8
S14	97.1	217.7	17.8	1.0	2.0	19.8	153.2	19.4	28.5	26.0	27.0	2.8
S21	96.9	221.1	17.8	1.0	10.4	20.2	156.5	20.4	28.4	26.1	27.3	2.8
S22	95.9	216.5	17.5	1.0	5.5	19.0	148.7	19.5	27.8	25.5	26.6	2.8
S23	100.2	225.9	18.2	1.0	16.2	19.8	166.8	20.1	29.0	26.6	27.7	2.9
S31	90.0	202.6	16.5	0.9	1.8	17.9	140.6	18.2	26.1	23.9	25.3	2.6
S32	89.8	199.8	16.1	0.9	1.8	17.5	139.7	17.8	25.8	23.6	25.2	2.6
S33	116.9	261.7	21.3	1.2	2.5	25.7	210.6	25.9	34.6	31.3	33.9	3.9
S41	90.7	201.0	16.6	5.7	1.9	18.4	706.5	19.2	31.4	25.5	25.6	2.6
S42	84.7	190.6	15.5	1.8	1.7	17.2	134.1	17.0	24.7	22.5	23.6	2.5
Taiyuan												
T11	104.2	234.5	19.1	2.6	12.5	21.0	505.6	21.0	31.4	28.4	29.5	3.1
T12	101.9	229.9	18.8	3.6	11.1	21.4	819.4	20.5	32.4	27.2	29.7	3.0
T13	152.8	336.4	27.6	1.5	19.2	29.9	1235.3	30.5	45.2	48.2	42.5	4.5
T21	95.6	213.4	17.3	1.0	36.9	19.2	214.2	19.1	27.6	25.5	27.1	2.8
T22	103.0	230.6	18.8	1.0	39.5	20.6	200.5	20.7	29.8	27.5	29.1	3.0
T23	104.0	234.2	19.2	1.0	49.3	20.9	164.5	21.1	30.4	27.8	29.3	3.0
T24	101.4	229.0	18.5	1.0	63.2	20.5	160.8	20.5	29.4	27.1	28.3	2.9
T31	103.8	232.9	19.3	4.0	10.5	22.1	2472.5	21.2	34.3	29.1	30.3	3.1
T32	220.8	406.5	45.5	14.7	29.6	196.6	2654.3	104.6	129.7	427.7	96.5	5.7
T33	97.5	222.1	18.3	8.6	6.8	39.9	2479.1	21.1	62.3	44.4	35.3	3.0
T34	99.9	225.1	18.8	5.1	4.2	26.1	1081.2	20.6	34.1	27.7	28.6	3.1
T41	104.4	233.3	21.2	14.5	7.5	72.1	282.9	24.5	61.9	72.8	34.4	3.5
T42	103.8	236.4	20.1	12.9	7.5	47.2	485.5	21.7	44.9	46.9	34.5	3.1
T43	106.7	221.0	21.3	7.1	12.6	53.8	3281.5	20.9	62.0	44.1	29.7	3.5
T44	87.6	203.5	16.2	3.4	3.7	20.0	294.8	18.4	29.3	24.5	24.7	2.5
Wuhan												
W11	58.8	132.4	11.6	0.6	4.4	12.0	411.5	11.8	17.3	15.8	16.7	1.7
W12	60.3	139.3	11.0	0.7	3.3	12.2	94.5	12.1	17.5	15.9	16.7	1.7
W13	60.2	134.6	11.1	0.7	4.8	11.9	93.3	12.1	17.4	15.8	16.7	1.8
W14	62.0	139.2	11.3	0.6	4.8	12.3	96.5	12.3	17.9	16.4	17.1	1.8
W21	55.2	123.0	10.1	0.5	26.0	10.9	87.2	10.9	15.8	14.6	15.3	1.6
W22	60.5	133.3	10.9	0.6	13.6	11.9	92.2	11.8	17.2	15.8	16.6	1.7
W23	61.7	138.4	11.3	0.6	28.6	12.3	113.4	12.4	17.9	16.3	17.4	1.8
W31	57.4	128.0	10.4	0.6	8.0	11.7	419.2	11.4	16.7	15.5	15.8	1.6
W32	29.8	66.6	5.4	0.3	3.9	6.0	312.9	6.1	8.7	8.2	8.5	0.9
W33	56.8	128.5	10.4	0.6	3.0	11.6	262.9	11.4	16.7	15.1	15.9	1.6
W34	29.6	66.5	5.4	0.7	2.8	6.0	290.1	5.9	9.4	8.4	8.8	0.9
W41	56.0	126.3	14.2	2.4	1.9	13.6	198.1	12.2	18.8	24.8	16.3	1.7
W42	59.7	133.4	11.3	2.9	1.3	11.9	1129.8	247.1	17.7	15.7	17.3	1.7
W43	67.4	138.6	14.8	0.8	7.6	24.4	353.5	34.2	36.9	26.4	26.5	2.6
Xinxiang												

X11	100.4	229.5	18.4	1.0	2.0	21.3	297.5	20.3	29.2	26.6	28.1	2.9
X12	101.4	231.0	18.5	1.0	2.8	20.7	197.5	21.0	29.1	27.8	29.0	2.9
X13	102.9	229.7	18.6	1.0	2.0	20.2	162.1	20.2	29.5	27.0	28.5	2.9
X21	101.8	228.7	18.4	1.0	3.9	20.4	157.6	20.3	29.3	26.8	28.0	2.9
X22	94.3	212.7	17.3	1.0	1.9	18.9	153.6	19.1	27.5	25.2	26.9	2.7
X23	96.7	218.4	17.7	1.0	2.0	19.4	153.9	19.9	28.3	25.8	27.2	2.8
X31	95.3	213.3	17.7	1.0	4.0	20.8	1841.3	21.1	32.3	54.8	30.2	2.9
X32	99.7	224.8	18.2	1.1	25.5	21.0	974.5	20.7	30.9	28.1	28.2	2.9
X41	95.8	213.8	17.9	1.2	2.2	19.6	1409.6	20.4	30.9	58.7	31.3	2.8
X42	102.9	223.2	18.8	10.1	2.3	24.7	2255.8	50.6	36.3	27.3	29.9	2.9
X43	100.8	228.4	19.5	16.5	5.5	21.5	210.3	22.9	34.8	27.6	29.1	3.2
X44	95.4	221.5	42.0	4.7	2.1	19.4	650.3	20.0	32.9	25.8	27.2	2.8

Table S3. List of target compounds and relative potency of individual PAHs compared with B[a]P (TEF values), according to different references

Compound	Abbr.	TEF.		
**parent-PAHs**				
Naphthalene	NAP	-	0.001[b]	0.001[c]
Acenaphthylene	AC	-	-	0.001[c]
Acenaphthene	ACE	-	0.001[b]	0.001[c]
Fluorene	FL	-	0.001[b]	0.001[c]
Phenanthrene	PHE	-	0.001[b]	0[c]
Anthracene	ANT	0.0005[a]	0.01[b]	0[c]
Fluoranthene	FLU	0.0005[a]	0.001[b]	0.08[c]
Pyrene	PYR	0.05[a]	0.001[b]	0[c]
Benz[a]anthracene	BaA	0.001[a]	0.1[b]	0.2[c]
Chrysene	CHR	0.005[a]	0.01[b]	0.1[c]
Benzo[b]fluoranthene	BbF	0.03[a]	0.1[b]	0.8[c]
Benzo[k]fluoranthene	BkF	0.2[a]	0.1[b]	0.03[c]
Benzo[a]pyrene	BaP	1[a]	1[b]	1[c]
Dibenz[a,h]anthracene	DBA	1[a]	1[b]	1[c]
Indeno[1,2,3-cd]pyrene	IP	0.05[a]	0.1[b]	0.07[c]
Benzo[g,h,i]perylene	BghiP	0.1[a]	0.01[b]	0.01[c]
**Nitro-PAHs**		**TEF.**		
1-Nitronaphthalene	1N-NAP	-		
2-Nitronaphthalene	2N-NAP	-		
5-nitroacenaphthene	5N-ACE	0.03 [d]		
2-Nitrofluorene	2N-FL	0.01 [d]		
3-Nitrophenanthrene	3N-PHE	-		
9-Nitrophenanthrene	9N-PHE	-		
2-Nitroanthracene	2N-ANT	-		
9-Nitroanthracene	9N-ANT	-		
3-Nitrofluoranthene	3N-FLU	-		
1-Nitropyrene	1N-PYR	0.1 [d]		
7-Nitrobenz(a)anthracene	7N-BaA	-		
6-Nitrochrysene	6N-CHR	10 [d]		

a Larsen JC, Larsen PB. 1998. Chemical carcinogens. In: AirPollution and Health (Hester RE, Harrison RM eds). Cambridge, UK: The Royal Society of Chemistry, 33-56.

b USEPA. 2010. Development of a Relative Potency Factor (RPF) Approach for Polycyclic Aromatic Hydrocarbon (PAH) Mixtures (External Review Draft). EPA/635/R-08/012A. Washington, DC.

c Schoeny R and Poirier K. 1993. Provisional Guidance for Quantitative Risk Assessment of Polycyclic Aromatic Hydrocarbons. EPA/600/R-93/089 (NTIS PB94116571). Washington, DC.

d Boström CE, Gerde P, Hanberg A, Jernström B, Johansson C, Kyrklund T, Rannug A, Törnqvist M, Victorin K, Westerholm R. 2002. Cancer risk assessment, indicators, and guidelines for polycyclic aromatic hydrocarbons in the ambient air. Environ Health Perspect, 110 Suppl 3:451-88.

Table S4. paticle-bound nitro-PAHs concentrations (pg/m3) at selected geographic locations.

name of site	nPAHs	range (pg/m3)	PM2.5/TSP	reference
Chinese cites	1n-Nap	29-400 (100 ± 34)	PM2.5	in this study
Chinese cites	2n-Nap	66-510 (220 ± 57)	PM2.5	in this study
Chinese cites	5n-Ace	5.4-56 (19 ± 6.3)	PM2.5	in this study
Chinese cites	2n-Fl	<1-35 (2.8 ± 4.8)	PM2.5	in this study
Chinese cites	9n-Phe	1.3-63 (7.6 ± 9.6)	PM2.5	in this study
Chinese cites	3n-Phe	6.0-200 (23 ± 18)	PM2.5	in this study
Chinese cites	2n-Ant	5.9-340 (36 ± 52)	PM2.5	in this study
Chinese cites	9n-Ant	87-6000 (560 ± 800)	PM2.5	in this study
Chinese cites	3n-Flu	8.7-160 (33 ± 19)	PM2.5	in this study
Chinese cites	1n-Pyr	8.2-430 (33 ± 37)	PM2.5	in this study
Chinese cites	7n-BaA	8.5-96 (29 ± 9.8)	PM2.5	in this study
Chinese cites	6n-Chr	<1-10 (2.9 ± 0.9)	PM2.5	in this study
Taiyuan, China	2n-Fl	19 ± 11	PM2.5	Li et al., 2014
Taiyuan, China	9n-Ant	118 ± 21	PM2.5	Li et al., 2014
Taiyuan, China	1n-Pyr	309 ± 135	PM2.5	Li et al., 2014
Northern Mexico city	9n-Phe	13.7 (pg/m3, Median)	PM2.5	Valle-Hernández et al, 2010
Northern Mexico city	3n-Phe	14.7 (pg/m3, Median)	PM2.5	Valle-Hernández et al, 2010
Northern Mexico city	9n-Ant	45.7 (pg/m3, Median)	PM2.5	Valle-Hernández et al, 2010
Northern Mexico city	2n-Flu	41.7 (pg/m3, Median)	PM2.5	Valle-Hernández et al, 2010
Northern Mexico city	3n-Flu	3.1 (pg/m3, Median)	PM2.5	Valle-Hernández et al, 2010
Northern Mexico city	1n-Pyr	10.9 (pg/m3, Median)	PM2.5	Valle-Hernández et al, 2010
Northern Mexico city	7n-BaA	16.3 (pg/m3, Median)	PM2.5	Valle-Hernández et al, 2010
Northern Mexico city	6n-Chr	1.7 (pg/m3, Median)	PM2.5	Valle-Hernández et al, 2010
Cordoba, Argentina	1n-Nap	17.29-18.96	TSP	Carreras, et al., 2013
Cordoba, Argentina	2n-Nap	15.89-17.43	TSP	Carreras, et al., 2013
Cordoba, Argentina	9n-Ant	28.07-56.50	TSP	Carreras, et al., 2013
Cordoba, Argentina	9n-Phe	21.04-42.80	TSP	Carreras, et al., 2013
Cordoba, Argentina	3n-Phe	30.69-45.09	TSP	Carreras, et al., 2013
Cordoba, Argentina	3n-Flu	25.36-27.81	TSP	Carreras, et al., 2013
Cordoba, Argentina	1n-Pyr	24.57-35.74	TSP	Carreras, et al., 2013
Cordoba, Argentina	7n-BaA	23.04-28.40	TSP	Carreras, et al., 2013
Cordoba, Argentina	6n-Chr	14.72-16.14	TSP	Carreras, et al., 2013
Marseilles area, France	1n-pyr	60.7(14.9-222.1)	TSP	Albinet et al., 2007
Marseilles area, France	7n-BaA	3.8 (0.2-9.9)	TSP	Albinet et al., 2007
Marseilles area, France	6n-Chr	33.1 (0.1-147.5)	TSP	Albinet et al., 2007

HCAB, Denmark	9n-Ant	63 ± 30	TSP	Feilberg et al., 2001
HCAB, Denmark	3n-Flu	39 ± 20	TSP	Feilberg et al., 2001
HCAB, Denmark	1n-Pyr	127 ± 44	TSP	Feilberg et al., 2001
Tokyo, Japan	1n-Pyr	8-36 fmol/m3	TSP	Kojima et al., 2010
Tokyo, Japan	2n-Flu	71-196 fmol/m3	TSP	Kojima et al., 2010
Madrid, Spain	3n-Phe	17.5 (7.4-46.6)	PM10	Barrado et al., 2013
Madrid, Spain	9n-Phe	19.8 (11.6-64.4)	PM10	Barrado et al., 2013
Madrid, Spain	1n-Pyr	41.4 (17.9-158.7)	PM10	Barrado et al., 2013
Madrid, Spain	3n-Flu	20.9 (9.2-63.5)	PM10	Barrado et al., 2013
Los Angeles, USA	9n-Ant	11-64	TSP	Reisen and Arey, 2005
Los Angeles, USA	1n-Pyr	3-38	TSP	Reisen and Arey, 2005
Roadside, UK	1n-Nap	2224 ± 2908	TSP	Alam et al., 2015
Roadside, UK	2n-Nap	1471 ± 1729	TSP	Alam et al., 2015
Roadside, UK	2n-Fl	689 ± 1034	TSP	Alam et al., 2015
Roadside, UK	9n-Ant	406 ± 595	TSP	Alam et al., 2015
Typical Motorbike city, Vietnam	2n-Ant	9.31	TSP	Thuy et al., 2012
Typical Motorbike city, Vietnam	9n-Ant	42-552	TSP	Thuy et al., 2012
Typical Motorbike city, Vietnam	1n-Pyr	29-488	TSP	Thuy et al., 2012
Typical Motorbike city, Vietnam	6n-Chr	125	TSP	Thuy et al., 2012
Typical Motorbike city, Vietnam	7n-BaA	41-214	TSP	Thuy et al., 2012
Sugar cane burning region	2n-Nap	nd-1800	TSP	Souza et al., 2014
Sugar cane burning region	9n-Phe	nd-6000	TSP	Souza et al., 2014
Sugar cane burning region	2n-Flu	nd-3400	TSP	Souza et al., 2014
Sugar cane burning region	3n-Flu	nd-610	TSP	Souza et al., 2014
Sugar cane burning region	7n-BaA	nd-1700	TSP	Souza et al., 2014

Refs

Li, R. J., Kou, X. J., Geng, H., Dong, C. & Cai, Z. W. Pollution characteristics of ambient PM2.5-bound PAHs and NPAHs in a typical winter time period in Taiyuan. Chinese Chemical Letters 25, 663-666 (2014).

Valle-Hernández, B. L. et al. Temporal variation of nitro-polycyclic aromatic hydrocarbons in PM10 and PM2.5 collected in Northern Mexico City. Sci. Total Environ. 408, 5429-5438 (2010).

Carreras, H. A., et al. omposition and mutagenicity of PAHs associated with urban airborne particles in Córdoba, Argentina. Environ. Pollut. 178, 403-410 (2013).

Albinet, A., Leoz-Garziandia, E., Budzinski, H. & Villenave, E. Polycyclic aromatic hydrocarbons (PAHs), nitrated PAHs and oxygenated PAHs in ambient air of the Marseilles area (South of France): Concentrations and sources. Sci. Total Environ. 384, 280-292 (2007).

Feilberg, A. B., Poulsen, M. W., Nielsen, T. & Henrik, S. Occurrence and sources of particulate nitro-polycyclic aromatic hydrocarbons in ambient air in Denmark. Atmos. Environ. 35, 353-366 (2001).

Kojima, Y. et al. Influence of secondary formation on atmospheric occurrences of oxygenated polycyclic aromatic hydrocarbons in airborne particles. Atmos. Environ. 44, 2873-2880 (2010).

Barrado, A. I., García, S., Castrillejo, Y. & Barrado, E. Exploratory data analysis of PAH, nitro-PAH and hydroxy-PAH concentrations in atmospheric PM10-bound aerosol particles. Correlations with physical and chemical factors. Atmos. Environ. 67, 385-393 (2013).

Reisen, F. & Arey, J. Atmospheric Reactions Influence Seasonal PAH and Nitro-PAH Concentrations in the Los Angeles Basin. Environ. Sci. Technol. 39, 64-73 (2005).

Alam, M. S. et al. Diurnal variability of polycyclic aromatic compound (PAC) concentrations: Relationship with meteorological conditions and inferred sources. Atmos. Environ. 122, 427-438 (2015).

Thuy, P. C., Kameda, T., Toriba, A., Tang, N. & Hayakawa, K. Characteristics of Atmospheric Polycyclic Aromatic Hydrocarbons and Nitropolycyclic Aromatic Hydrocarbons in Hanoi-Vietnam, as a Typical Motorbike City. Polycyclic Aromatic Compounds 32, 296-312 (2012).

Souza, K. F., Carvalho, L. R. F., Allen, A. G. & Cardoso, A. A. Diurnal and nocturnal measurements of PAH, nitro-PAH, and oxy-PAH compounds in atmospheric particulate matter of a sugar cane burning region. Atmos. Environ. 83, 193-201 (2014).

Figure S1. Percentage composition of 2-, 3-, 4-, 5- and 6- ring PAHs in PM2.5 collected from each site.

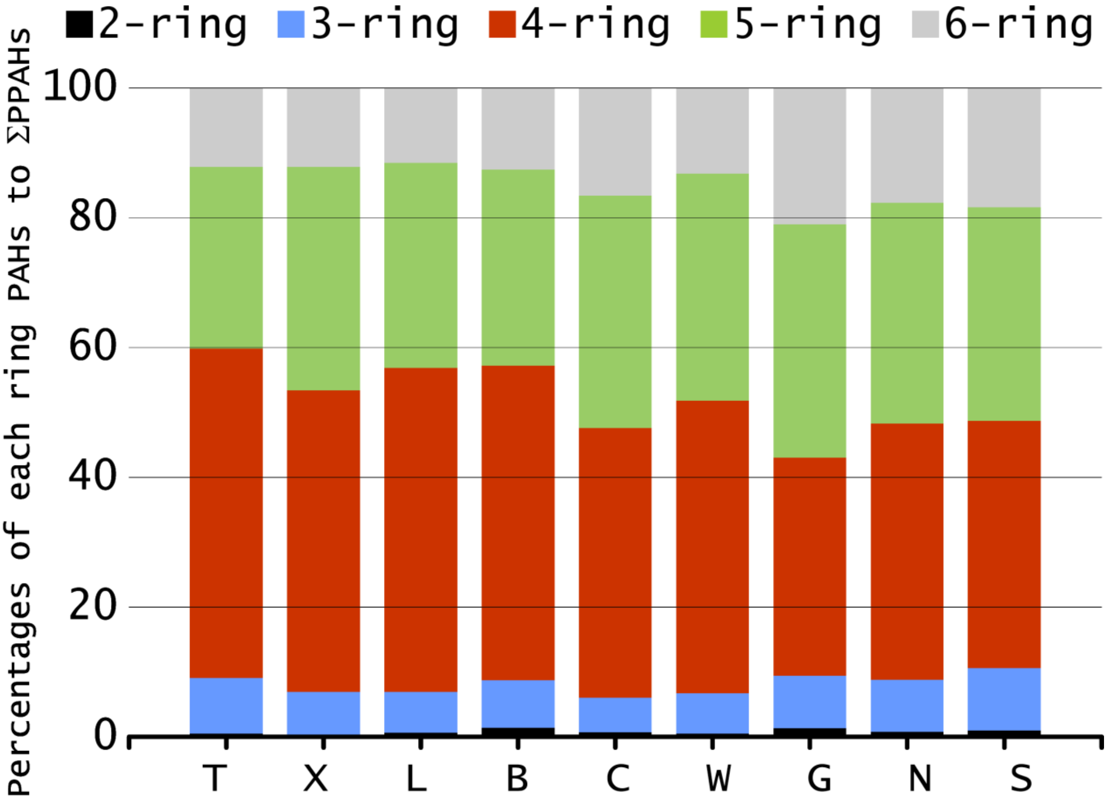

Figure S2. The relationships between the PMF predicted/modeled Q values for 4-factors, 5-factors and 6-factors results.

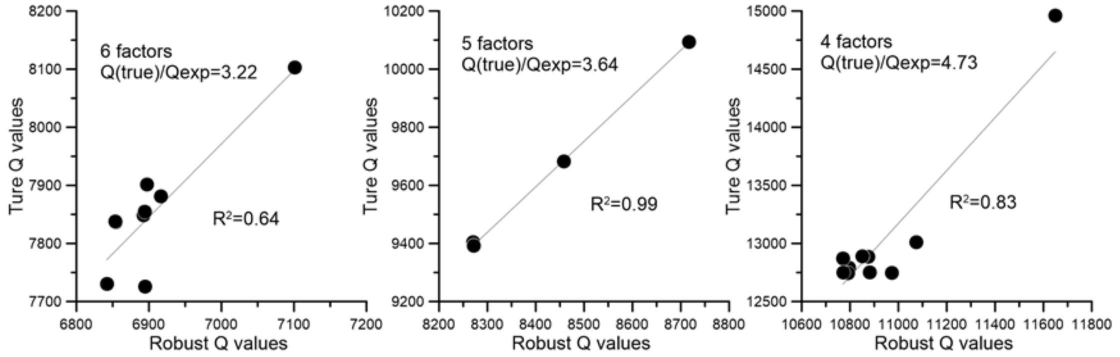

Figure S3. 5-factor loadings by PMF analysis from parent PAHs and nitro-PAHs data of PM2.5 sample at nine urban sites across China.

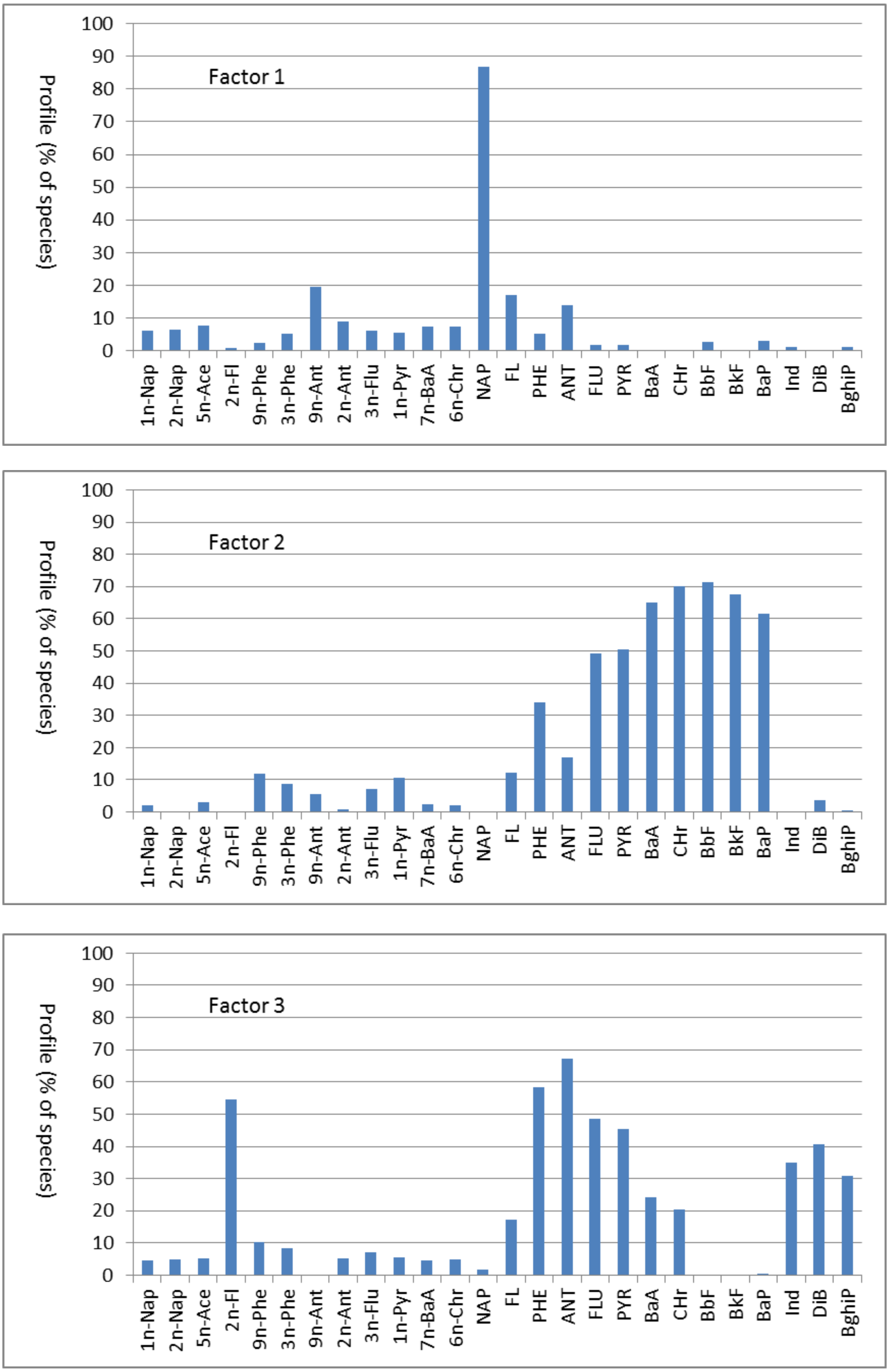

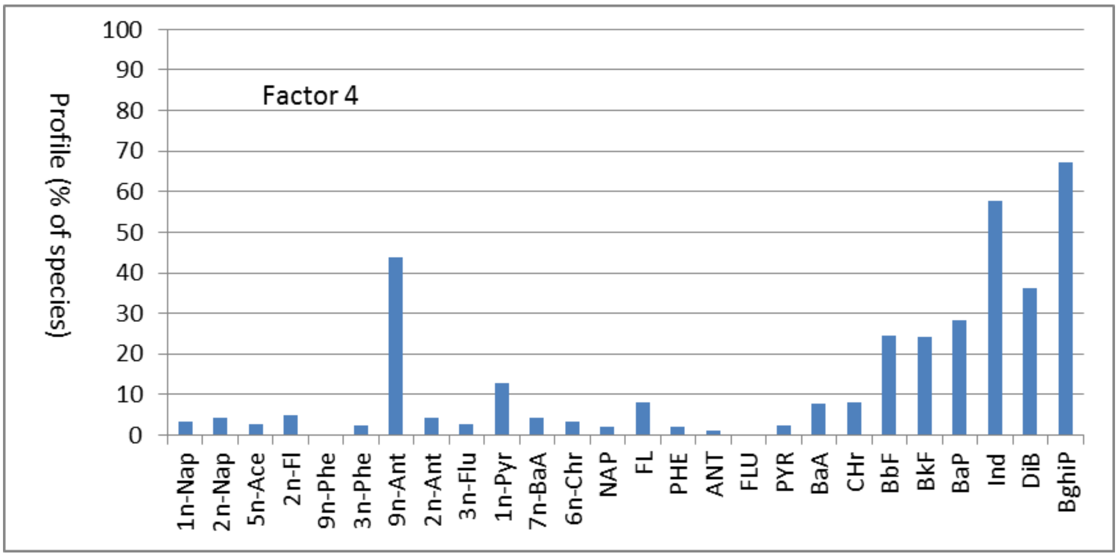
Factor 4
Profile (% of species)
100
90
80
70
60
50
40
30
20
10
0
1n-Nap
2n-Nap
5n-Ace
2n-Fl
9n-Phe
3n-Phe
9n-Ant
2n-Ant
3n-Flu
1n-Pyr
7n-BaA
6n-Chr
NAP
FL
PHE
ANT
FLU
PYR
BaA
CHr
BbF
BkF
BaP
Ind
DiB
BghiP

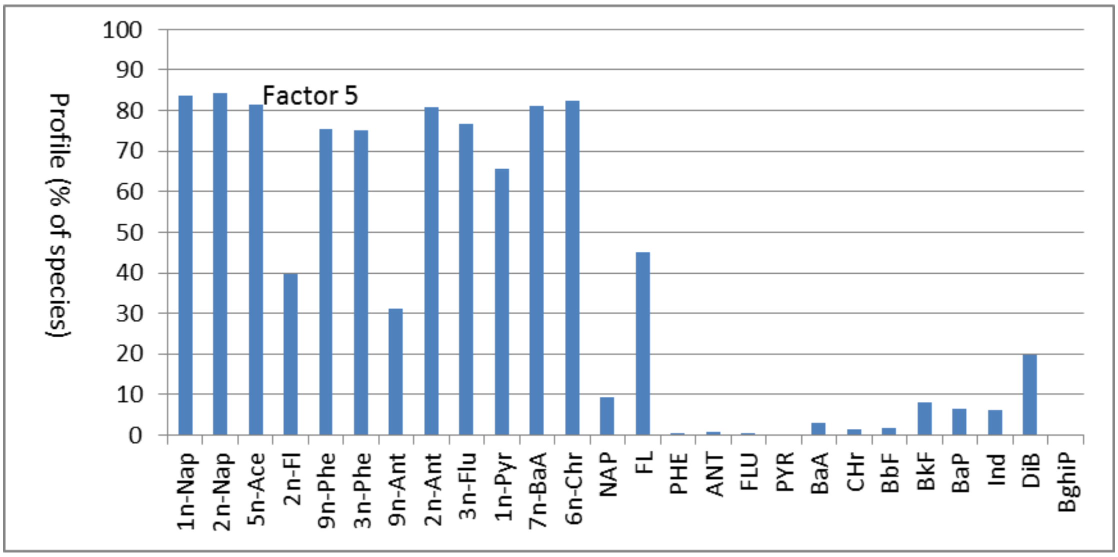
Factor 5
Profile (% of species)
100
90
80
70
60
50
40
30
20
10
0
1n-Nap
2n-Nap
5n-Ace
2n-Fl
9n-Phe
3n-Phe
9n-Ant
2n-Ant
3n-Flu
1n-Pyr
7n-BaA
6n-Chr
NAP
FL
PHE
ANT
FLU
PYR
BaA
CHr
BbF
BkF
BaP
Ind
DiB
BghiP

Figure S4. 4-factor loadings by PMF analysis from parent PAHs and nitro-PAHs data of PM2.5 sample at nine urban sites across China.

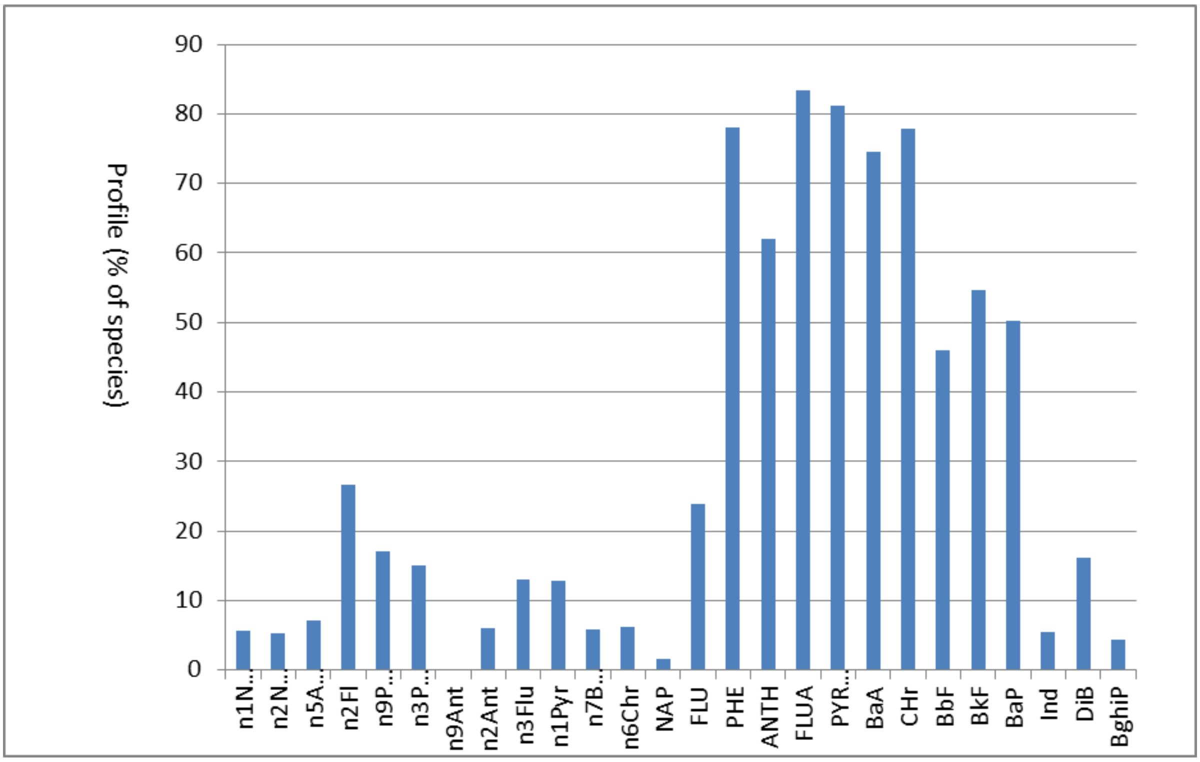

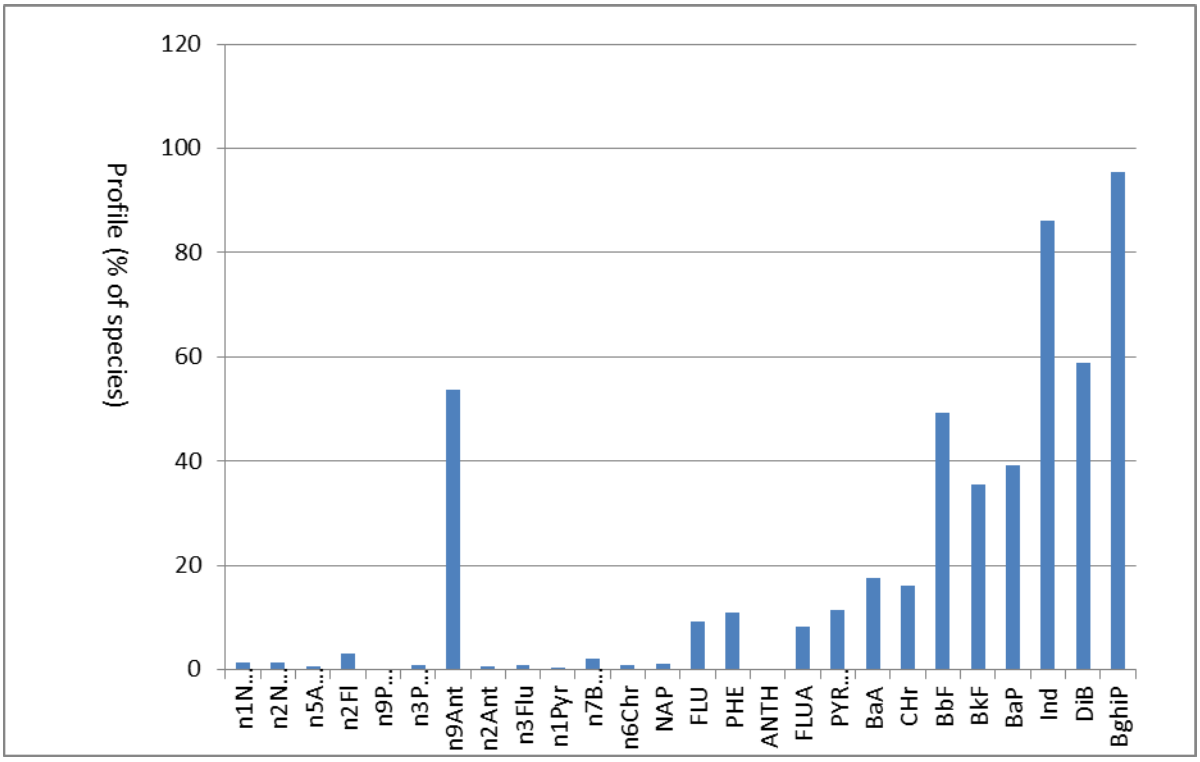

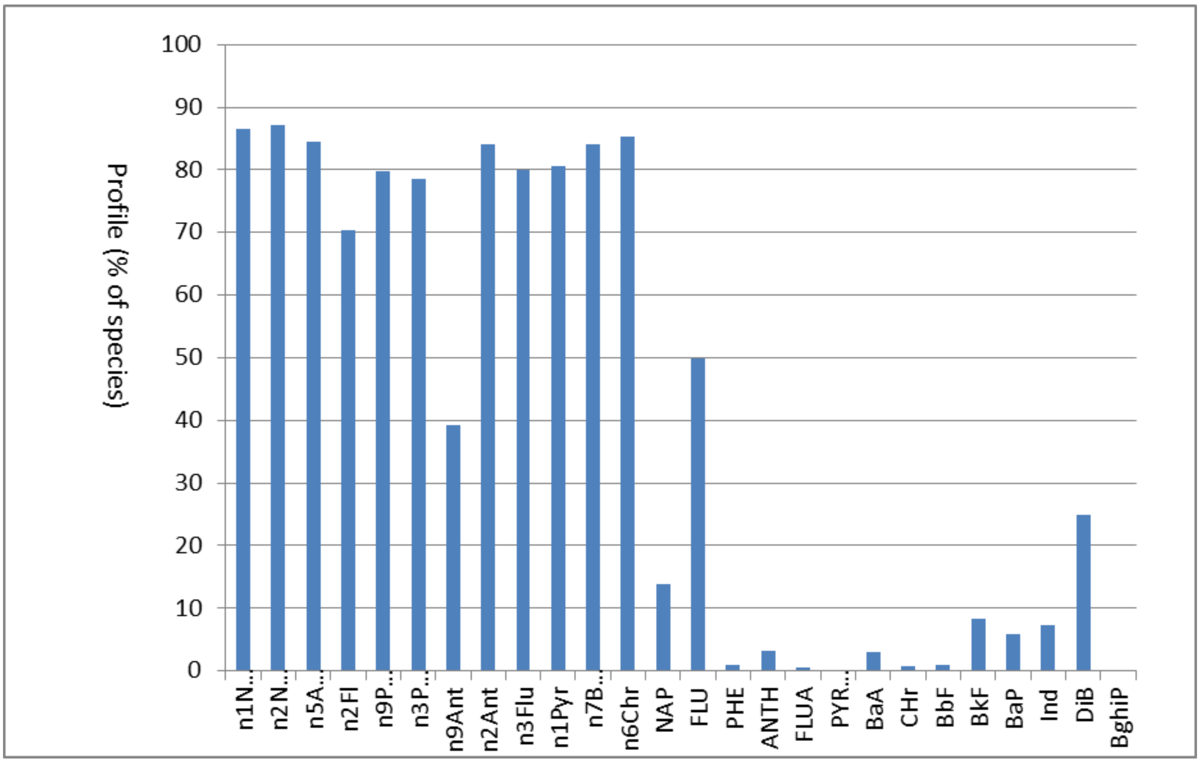
Profile (% of species)
100
90
80
70
60
50
40
30
20
10
0
n1N…
n2N…
n5A…
n2Fl
n9P…
n3P…
n9Ant
n2Ant
n3Flu
n1Pyr
n7B…
n6Chr
NAP
FLU
PHE
ANTH
FLUA
PYR…
BaA
CHr
BbF
BkF
BaP
Ind
DiB
BghiP

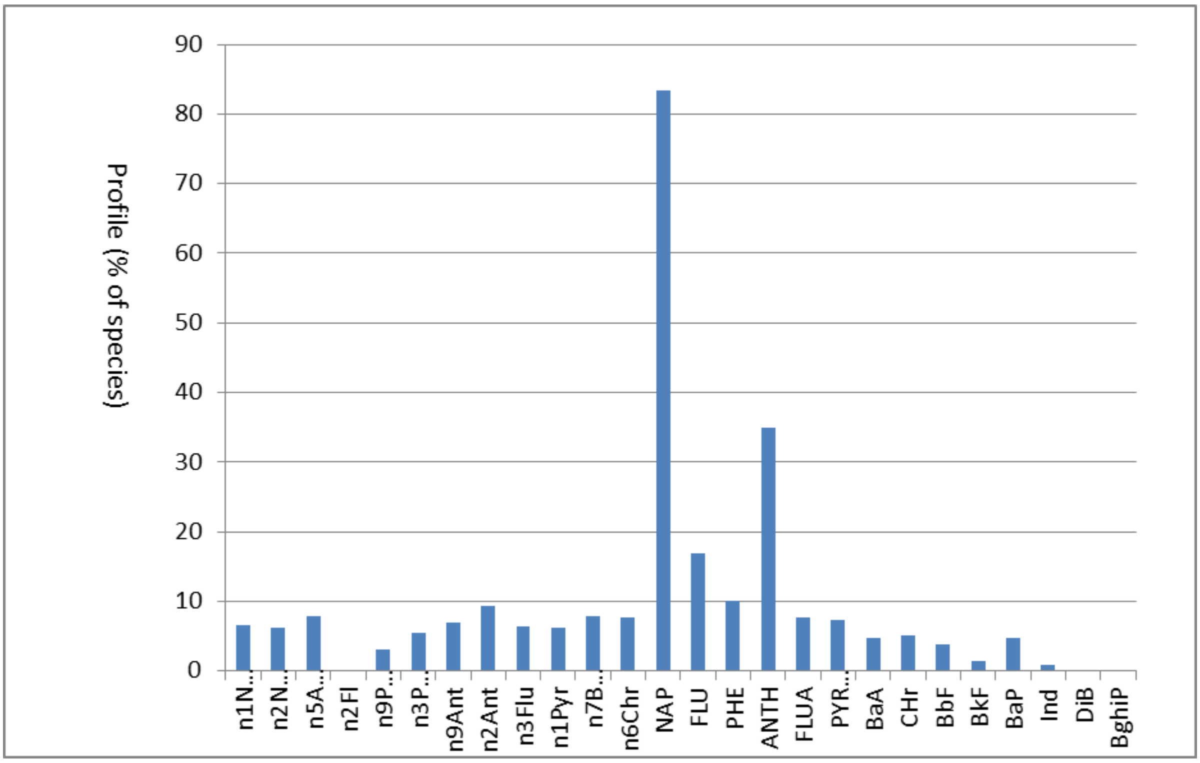
Profile (% of species)
90
80
70
60
50
40
30
20
10
0
n1N…
n2N…
n5A…
n2Fl
n9P…
n3P…
n9Ant
n2Ant
n3Flu
n1Pyr
n7B…
n6Chr
NAP
FLU
PHE
ANTH
FLUA
PYR…
BaA
CHr
BbF
BkF
BaP
Ind
DiB
BghiP

Figure S5. 6-factor loadings by PMF analysis from parent PAHs and nitro-PAHs data of PM2.5 sample at nine urban sites across China.

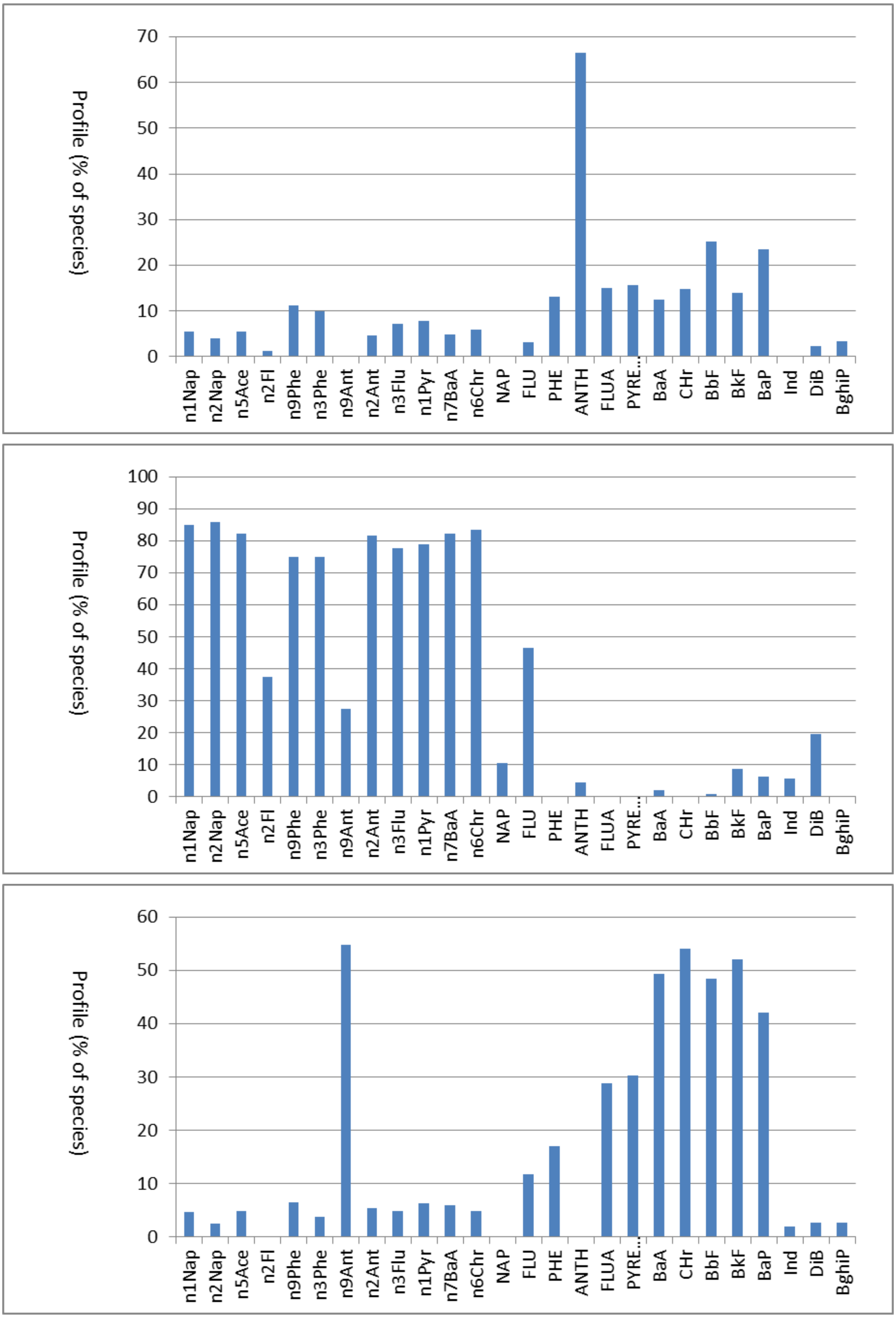

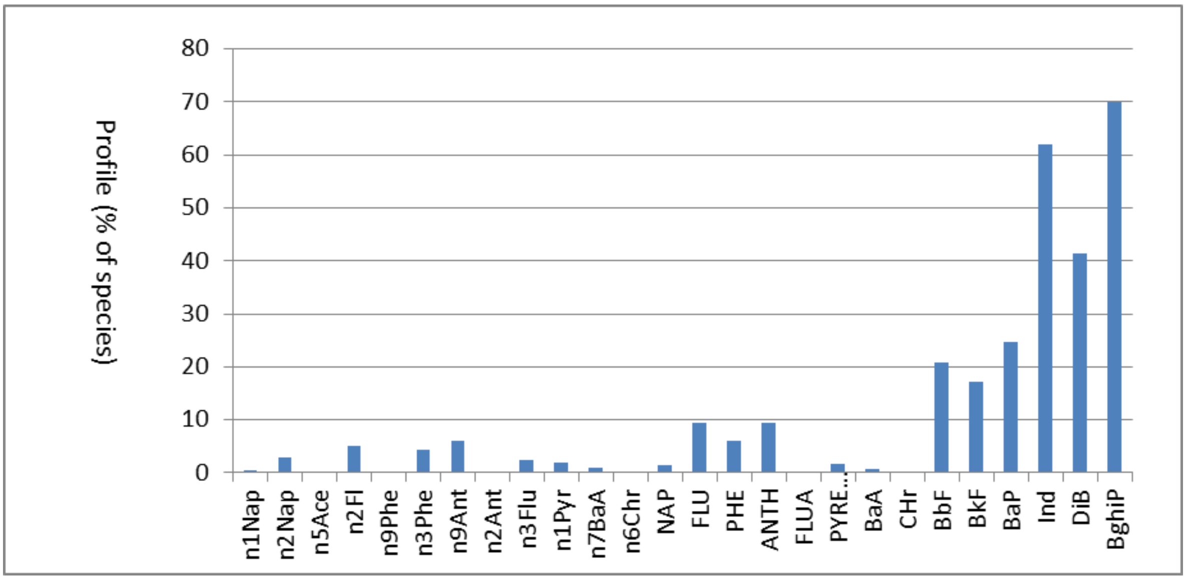
Profile (% of species)
0
10
20
30
40
50
60
70
80
n1Nap
n2Nap
n5Ace
n2Fl
n9Phe
n3Phe
n9Ant
n2Ant
n3Flu
n1Pyr
n7BaA
n6Chr
NAP
FLU
PHE
ANTH
FLUA
PYRE...
BaA
CHr
BbF
BkF
BaP
Ind
DiB
BghiP

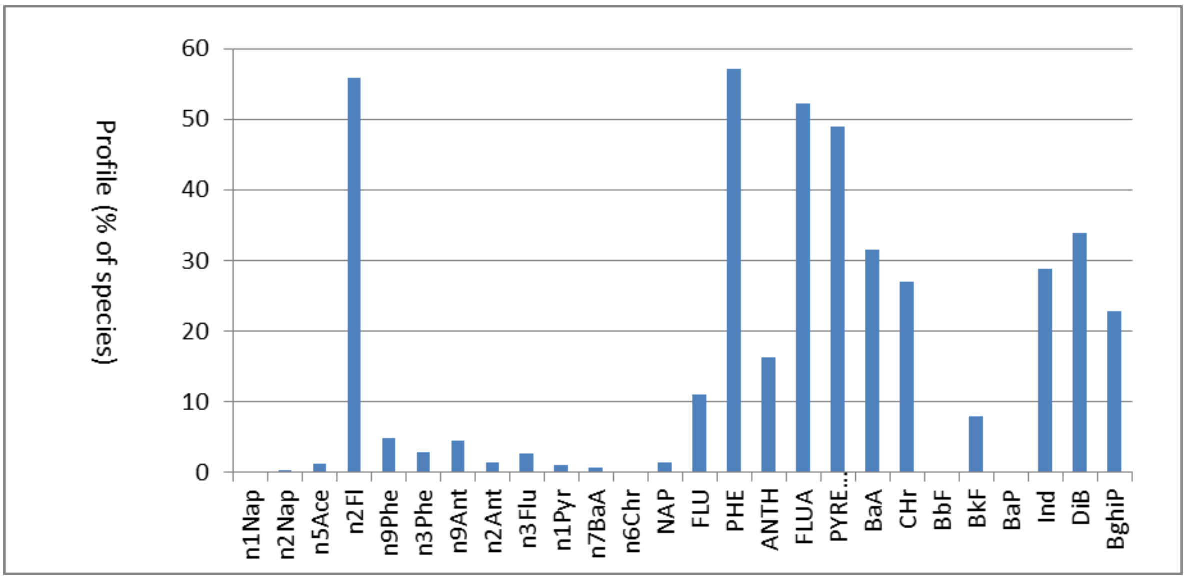
Profile (% of species)
0
10
20
30
40
50
60
n1Nap
n2Nap
n5Ace
n2Fl
n9Phe
n3Phe
n9Ant
n2Ant
n3Flu
n1Pyr
n7BaA
n6Chr
NAP
FLU
PHE
ANTH
FLUA
PYRE...
BaA
CHr
BbF
BkF
BaP
Ind
DiB
BghiP

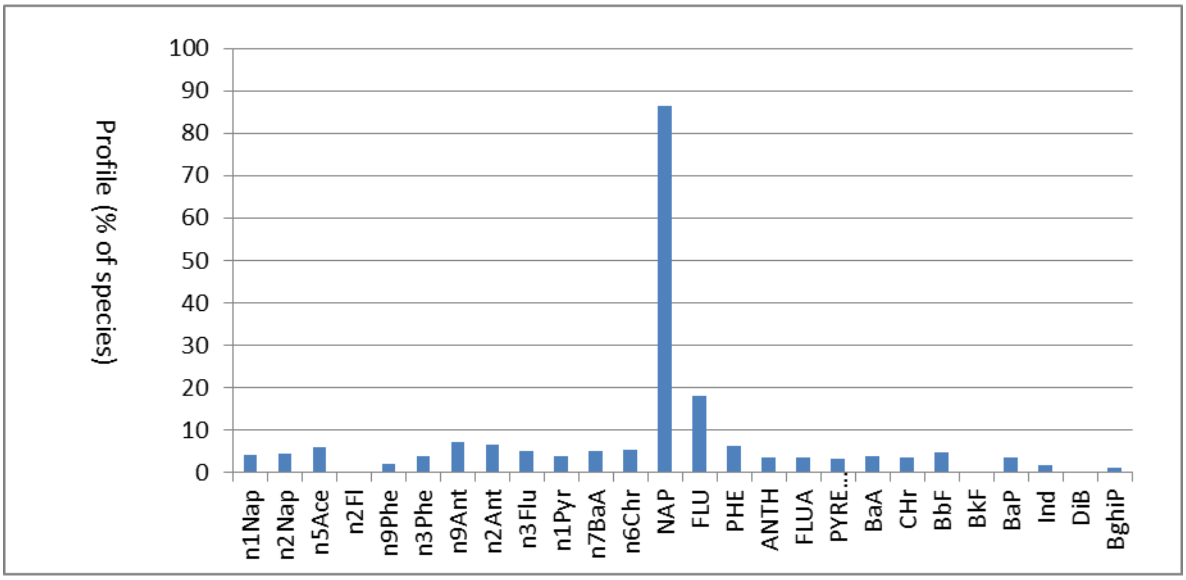
Profile (% of species)
0
10
20
30
40
50
60
70
80
90
100
n1Nap
n2Nap
n5Ace
n2Fl
n9Phe
n3Phe
n9Ant
n2Ant
n3Flu
n1Pyr
n7BaA
n6Chr
NAP
FLU
PHE
ANTH
FLUA
PYRE...
BaA
CHr
BbF
BkF
BaP
Ind
DiB
BghiP
